# Inherently-Forced Tensile Strain in Nanodiamond-Derived Onion-like Carbon: Consequences in Defect-Induced Electrochemical Activation

**DOI:** 10.1038/srep23913

**Published:** 2016-04-01

**Authors:** Young-Jin Ko, Jung-Min Cho, Inho Kim, Doo Seok Jeong, Kyeong-Seok Lee, Jong-Keuk Park, Young-Joon Baik, Heon-Jin Choi, Seung-Cheol Lee, Wook-Seong Lee

**Affiliations:** 1 Center for Electronic Materials, Korea Institute of Science and Technology, Seoul 136-791, Republic of Korea; 2 Department of Materials Science and Engineering, Yonsei University, 262 Seongsanno, Seodaemun-Gu, Seoul 120-749, Republic of Korea; 3 Indo-Korea Science and Technology Center, Korea Institute of Science and Technology, Bangalore 560064, INDIA

## Abstract

We analyzed the nanodiamond-derived onion-like carbon (OLC) as function of synthesis temperature (1000~1400 °C), by high-resolution electron microscopy, electron energy loss spectroscopy, visible-Raman spectroscopy, ultraviolet photoemission spectroscopy, impedance spectroscopy, cyclic voltammetry and differential pulse voltammetry. The temperature dependences of the obtained properties (averaged particle size, tensile strain, defect density, density of states, electron transfer kinetics, and electrochemical oxidation current) unanimously coincided: they initially increased and saturated at 1200 °C. It was attributed to the inherent tensile strains arising from (1) the volume expansion associated with the layer-wise diamond-to-graphite transformation of the core, which caused forced dilation of the outer shells during their thermal synthesis; (2) the extreme curvature of the shells. The former origin was dominant over the latter at the outermost shell, of which the relevant evolution in defect density, DOS and electron transfer kinetics determined the electrochemical performances. In detection of dopamine (DA), uric acid (UA) and ascorbic acid (AA) using the OLC as electrode, their oxidation peak currents were enhanced by factors of 15~60 with annealing temperature. Their limit of detection and the linear range of detection, in the post-treatment-free condition, were as excellent as those of the nano-carbon electrodes post-treated by Pt-decoration, N-doping, plasma, or polymer.

For a wide variety of the nano-carbon (n-C) materials applications, the defect control is an important issue^1^. While in many cases one requires defect minimization or healing, in some other cases the opposite is true. For example, the electrochemical performance of graphene, *e.g.* as the electrochemical electrode, is enhanced by their defects that generate mid-gap states, which accelerate the electron tunneling through the electrode-solution interface^2^. While the defect generations in graphene had been enabled by such post-treatments as the plasma treatments^3^, e-beam irradiation^4^, or doping^5^, the efforts should be minimized. Thermal activation is a desirable alternative technique, since it might be autonomously achieved within a thermal synthesis cycle as for the nanodiamond-derived onion-like carbon (OLC)^6^, apart from its advantage of the simplicity and facile mass-production. Unfortunately, the defects in many n-C materials, *e.g.* graphene, graphene nanoribbon, carbon nanotubes, are thermally *healed*, rather than generated (Table S1)^7^,^8^,^9^,^10^,^11^ due to their high vacancy formation energy^12^ in a wide temperature range encompassing up to 3000 °C.

By contrast, the relevant reports on OLC varied depending on the adopted temperatures: defects generation at relatively low temperatures^13^,^14^, in costrast to the defect healing at higher temperatures (Table S1)^14^,^15^,^16^. Some report was even contradictory to other reports (Table S1)^17^. Moreover, the origin of the annealing-induced defect density evolution in OLC, relevant to its electrochemical performance, is still far from clarified; here, we investigated this issue, for OLC produced by vacuum annealing of detonation nanodiamond^6^, at relatively low annealing temperatures of 1000–1400 °C. While the OLC had been actively investigated for the capacitive energy storage device, the study on its application for electrochemical detection of some important biomolecules such as dopamine (DA), uric acid (UA) and ascorbic acid (AA), is in its infant stage^18^. Their reported performance was relatively poor compared to those of other n-C-based electrode materials^19^,^20^, even though it was additionally post-treated by polymer^18^. Here we demonstrated that, even without further post-treatments, the performance of the present OLC electrode optimized within the thermal synthesis cycle, was as excellent as those of the various post-treated n-C electrodes.

## Results and Discussion

**Figure 1:**
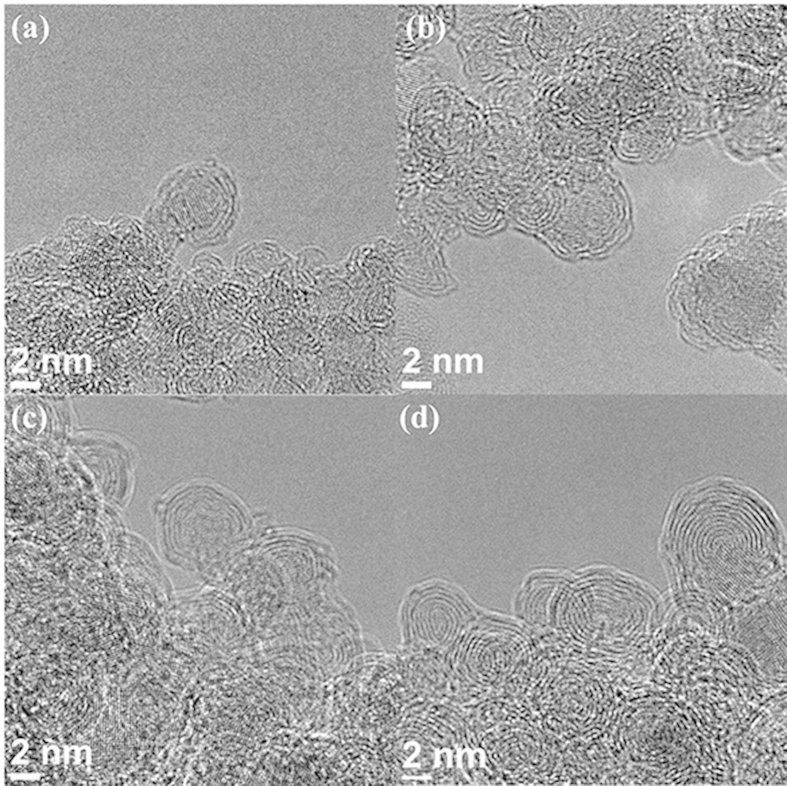
HR-TEM images of OLCs synthesized by annealing the detonation nanodiamond at temperatures of (**a**) 1000 °C, (**b**) 1100 °C, (**c**) 1200 °C and (**d**) 1400 °C.

**Figure 2:**
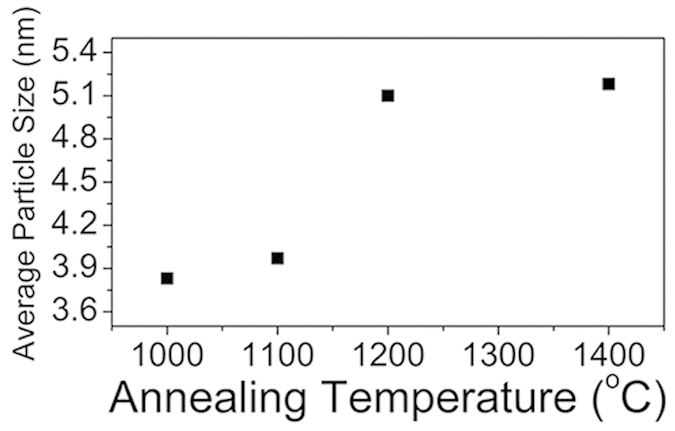
The average particle size vs annealing temperature profile of the OLCs. 10~20 HR-TEM pictures were taken for each OLC sample, from which 50–55 clearly isolated particles (See Fig. S4) were selected for the particle size measurement for each OLC sample. See Fig. S5 where the particle size distributions of each OLC samples were given.

Figure 1 shows the HR-TEM pictures of the nanoparticles synthesized by annealing the detonation nanodiamond powder at various temperatures. The core-shell structures of the particles were obvious, with the diamond {111} planes at the core (with interplanar spacing of 0.21 nm) and the approximately concentric graphitic shells; their interplanar spacing varied from 0.34 nm to 0.29 nm (see for example, Fig. S2 and Table S2 for OLC-1400); they decreased with their radial distance from the particle surface, in accordance with the previous reports^21^. The XRD pattern (Fig. S3) also revealed the coexistence of the diamond peaks (at 2θ = 43.9, 75.3) and the graphite peak (at 2θ = 26.4), *e.g.* for OLC-1200 sample. The number of shells increased with temperature, at the expense of the diamond core, which almost vanished at 1400 °C (Fig. 1d, see Statement S1 for the naming of the particle). Figure 2 shows the temperature-dependence of the averaged OLC particle diameter. It initially increased with temperature and was eventually started to saturate at 1200 °C. The increasing OLC particle size was attributed to the observation that the interplanar spacing of the newly-generated graphitic shells was about 50% larger than that of the {111} diamond planes at the core, as stated above. It was further supported by the well-known fact that the graphite-to-diamond phase transformation could proceed via the puckering of the graphite basal planes to transform into diamond {111} planes, thereby reducing their interplanar spacing^22^; it indicated that the reverse process would increase the interplanar spacing as in the present case. Additional possible particle growth mechanisms will be discussed later.

The consecutive temperature-dependent microstructural variations of the core-shell structure (Fig. 1) suggested the analogous isothermal transformation from surface to the core, as reported previously (Kuznetsov *et al.*^23^, Pech *et al.*^6^: see Fig. S1); it was further supported by the HR-TEM picture of the OLC-1200 after zero-time annealing at 1200 °C (Fig. S6: HR-TEM picture; Fig. S7: particle size distribution), which was rather similar to the as-received nanodiamond powder (Fig. S1a). It indicated the obvious isothermal volume expansion after 1 hour annealing for the OLC-1200. It strongly suggested that the diamond core, encapsulated by the new-born outer shells, would subsequently undergo additional diamond-to-graphite transformations to generate the inner shells, of which the associated volume expansion should eventually cause the forced radial expansion of the outermost shells *i.e.* the forced tensile strain in tangential direction. Such process would continue until the diamond core is completely consumed. Due to the action-reaction principle, such forced radial expansion of the outer shells by the new-born inner shells would result in the corresponding radial compression of the inner shells. Such picture was supported by the radially decreasing inter-shell spacing observed at *e.g.* OLC-1400 (Fig. S2 and Table S2). It was also in accord with the previous reports^21^, where the focus was on the cumulative radial compression of the inner shells to generate a high pressure and consequent *graphite-to-diamond* transformation at the *core* in OLC derived from the e-beam-irradiated amorphous carbon. By contrast, here we focus on the reverse reaction *i.e.* the *diamond-to-graphite* transformation at the core to generate the inner shells, which might drive the radial expansion and possibly the consequent tangential tensile strain of the outer shells. Nevertheless, it is important that there is another possible origin of the strain, *i.e.* the shell curvature which was orders of magnitude greater than the flat graphene layers with mild corrugations^24^; it might lead to the accordingly large tensile strain in the shells, as previously studied for the graphene nano-bubble^25^.

**Figure 3:**
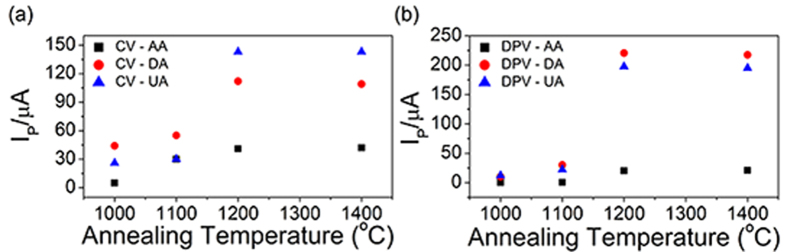
The peak current *vs* annealing temperature profiles of the OLC electrodes corresponding to the (**a**) CV profiles shown in Fig. S8a–c and (**b**) DPV profiles shown in the Fig. S9.

Such hypothetical tensile strain in the outer shells might result in the defect generation, which might alter the mid-gap state and the electrochemical activity^2^. To test the possible effect of such hypothetical strain on the defect formation and consequent electrochemical performances, the OLCs described in Fig. 1 were drop-coated on the glassy carbon electrode for individual electrochemical detection of AA, DA, and UA, employing the cyclic voltammetry (CV) and differential pulse voltammetry (DPV). The resulting CV responses were given in Fig. S8a–c, of which the peak currents and peak potentials were summarized in Table 1. As shown in Fig. 3a, the peak currents initially increased by factors of 2.5 (DA), 4.5 (UA), 8 (AA) (Table 1) with temperature at 1000~1200 °C, and were saturated thereafter. Figure S8d shows the CV responses of OLC electrodes from the mixed solution containing 0.33 mM of the three analytes, respectively. Oxidation peak positions did not differ much from those of their individual CV responses, which indicated that the mutual interference was not significant. Furthermore, the annealing-temperature dependence of the peak currents for the simultaneous detection was similar to that of the individual detections.

Figure S9 shows the DPV responses from the OLC electrodes for the *mixture* solution of the three analytes. Figure 3b shows the temperature-dependences of the peak currents. They closely resembled the case of CV (Fig. 3a): they increased by factors as large as 20 (DA), 15 (UA) and 60 (AA) (Table 1) in the range of 1000~1200 °C and were saturated subsequently. Furthermore, the peak separations in the DPV response from the OLC-1200 sample (Table S3), obtained from Fig. S9, were similar to those of other n-C materials post-treated in various ways (see Statement S2 for further comments). While such electrode-material-dependence suggested the relevant rate-determining step of electron transfer at electrode surface, an additional step should be considered also: the analyte diffusion in the solution^26^. Indeed, the further inspecting the CV responses (see Figs S10, S11 and the related Statement S3) indicated that the oxidation reaction was *mixed*-controlled.

**Figure 4:**
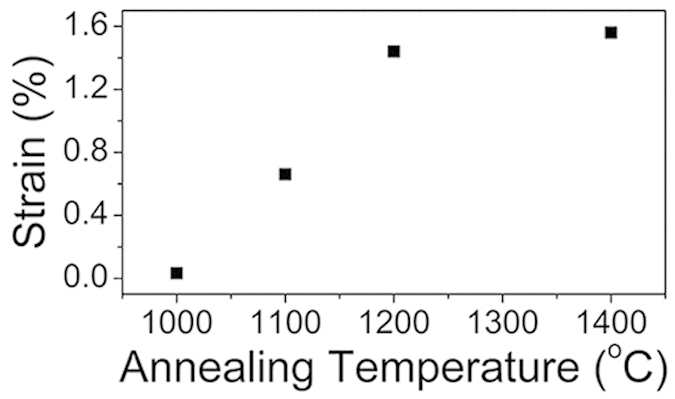
Strain of OLC nanoparticles estimated from the shift in 2D peak frequency of Raman spectra, relative to that of the flat multilayer graphene (MLG); the effect of the number of layers on the 2D band peak frequency, for the flat MLG, was included in the calculation^30^.

The electron-transfer kinetics depends upon the mid-gap states of which the DOS is enhanced by the defect generation in the graphene electrode^2^. While plasma treatment^27^, e-beam irradiation^28^ or nitrogen doping^19^ were adopted for defect generations in graphene, it was also enabled by the curvature-induced strain in the graphene nano-bubbles^24^,^25^, of which the radii were similar to those of the OLCs in the present study (Fig. 1). It prompted us for the analogous analyses. Figure S12a,b compared the Raman spectra obtained from the present OLC samples. The D/G band peak positions and the peak intensity ratios were summarized in Table S4, along with the 2D band peak positions. The latter resided in the range of 2635~2676 cm^−1^, which were well below that of the flat, unstressed monolayer graphene (2678 cm^−1^)^25^; it indicated the *tensile* strain in the shells according to the previous report^29^. Furthermore, since the increasing number of graphene layers is known to cause a *blue-shift* in the peak frequency of 2D band^30^, the actual strain of the present multi-shell OLC samples might be even greater than that inferred above (Table S4). Figure 4 shows the temperature dependence of the tensile strain inferred from the aforementioned analyses.

**Figure 5:**
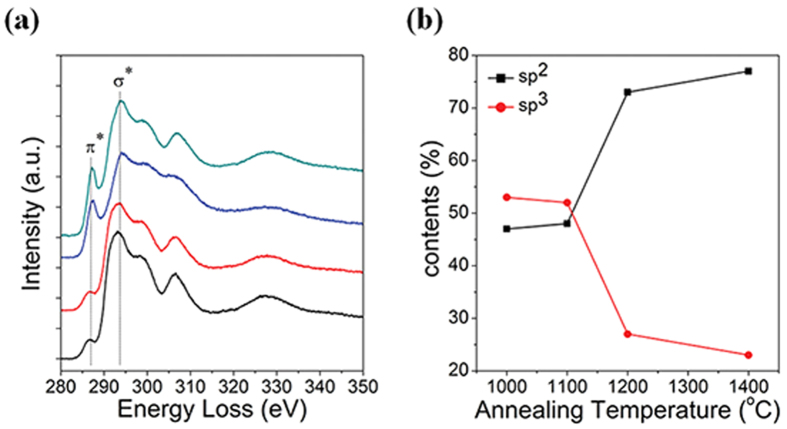
(**a**) EELS profiles of OLC samples (black: OLC-1000, red: OLC-1100, blue: OLC-1200, dark cyan: OLC-1400) and (**b**) The annealing-temperature dependence of sp^2^ and sp^3^ contents corresponding to EELS profiles **(a)**.

**Figure 6:**
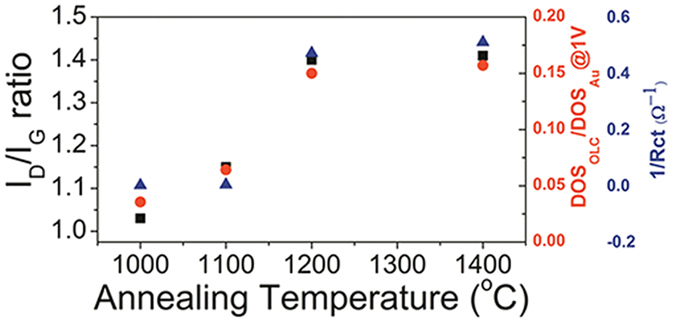
The annealing-temperature dependences of the I_D_/I_G_ ratio, DOS, and Rct^−1^ corresponding to Figs S14, S15 and S18.

The drastic annealing-induced enhancement in electrochemical responses (Fig. 3) suggested relevant changes in defect density and DOS; the former was analyzed from the D to G band intensity ratio (I_D_/I_G_) in Raman spectra, which is well known to represent the defects density^30^,^31^. It actually increased with the annealing-temperature (Figs S12a and S14, Table S4); the effect of the detonation nanodiamond on the Raman spectra was negligible as shown in Fig. S13 and in its caption. Consequent change in DOS was analyzed by UPS (Figs S12c,d, and S15). The inverse of the charge transfer resistance (Rct^−1^), which might translate to the charge transfer kinetics, was obtained by the impedence spectroscopy (Figs S16, S17 and S18, Table S5 and Statement S4). Furthermore, since the core (diamond) versus the shell (graphite) masses were important for the defect generation mechanism in later discussions, it was analysed by EELS (as the sp^2^/sp^3^ ratio) as shown in Fig. 5. The I_D_/I_G_ ratio, DOS, and Rct^−1^ were compared in a graph (Fig. 6). It was remarkable that their temperature dependences *unanimously* agreed among themselves, as well as with those of OLC particle size (Fig. 2), the electrochemical responses (Fig. 3), and the strain (Fig. 4), in the initial increase and the eventual saturation at 1200 °C. It is worth to note here that the defect density (I_D_/I_G_ ratio in Fig. 6), strain (Fig. 4) and the sp^2^/sp^3^ carbon contents (Fig. 5) originate from all the shells of the particle due to the penetration depths of Raman spectroscopy and EELS (Experimental details) which were much larger than the OLC radii. It was in contrast to the electrochemical performances (Fig. 3) and Rct^−1^ (Fig. S18), which should originate exclusively from the outermost shell, since the electron transfer to the inner shells is unlikely, not only due to the electron interceptions by the acceptor states in the intervening outermost shell but also due to the exponentially decreasing tunneling probability with respect to the tunneling distance^32^. The DOS data (Fig. S15) is also outermost-shell-specific, due to the short penetration depth of UPS (Experimental details).

The aforementioned unanimous agreement strongly suggested some common underlying origin. To examine it, let us first focus on the possible correlation between the strain (Fig. 4) and the defect density (*i.e.* the I_D_/I_G_ ratio: Fig. 6), in the light of the vacancy formation energy (Ev) versus the atom migration energy barrier (Em) of the carbon materials reported previously (Table S6). The lower Ev indicated easier vacancy formation, while the lower Em indicated the easier atomic migrations around the defects and hence possibly their healing. An interesting effect of the molecular curvature on Ev and Em was disclosed from Table S6. The Ev (for single vacancy) decreased while the Em increased in the order of graphene, SWCNT, and fullerene, *i.e.* in the order of their molecular curvatures. It strongly suggested possible effect of the strains (probably induced by the molecular curvature) on Ev; at very large strain (irrespective of the source of the strain), the Ev might be accordingly altered so that the vacancy generation might be accelerated relative to its healing. We will clarify such possibility for the present OLC samples as follows.

As our OLC sample were found to be under the inherent *tensile* strain as shown above, a bond between a given pair of carbon atoms should have been stretched, accordingly closer to the threshold rupture strain which is known to be 6~8% for graphene^33^. Apart from such inherent tensile strain, additional tensile strain might be imposed on the bond by the thermal vibrations of the bound atoms at the annealing temperatures. The collective thermal motions of the atoms in crystalline solid is governed by the Debye model^34^, where the phonon modes distribution would obviously shift to the higher energy domain with temperature. It would lead to greater chances of the thermally-triggered rupture of the bonds, provided that the inherent tensile strain had already brought the bond close enough to the rupture threshold. Such possibility was actually supported by a recent works on the thermally facilitated rupture of the mechanically strained polycrystalline graphene in the temperature range encompassing up to 1200 °C (section 3.3 of the reference^35^). It was also supported by the annealing-induced dangling bond generation at the nanodiamond-derived OLC in a previous report^15^ in the temperature range including that of present experiment; since the vacancy generation was known to generate dangling bonds in the OLC^36^, it suggested accordingly enhanced vacancy generation. Such conclusion was further supported by the reports by Okotrub *et al.*^37^, where they confirmed the thermally-induced void formation in nanodiamond-derived OLC by x-ray emission spectroscopy, although the relevant temperature was higher than that in the present study. Nevertheless, such temperature dependence contradicted the thermal *healing* of the defects reported in other forms of n-Cs, or in OLC at relatively high temperature range (Table S1). The aforementioned considerations concerning Ev and Em suggested that the defect generations/healing might mutually compete, and that their relative dominances might depend on the magnitude of the inherent strain of the molecular bonds. Indeed, when the strain is not large enough, the thermally-triggered rupture would be negligible even for the same phonon mode distributions; the thermal motions would just suffice for the thermal migration around the defects and consequent healing.

How would these two strain sources, *i.e.* the core volume expansion and the shell curvature, be correlated to the aforementioned temperature-dependences of the wide variety of properties, respectively or collectively? Recall that the electron transfer to the outermost shell dominated the electrochemical performance (Fig. 3) and the Rct^−1^ (Fig. S18). The UPS-derived DOS (Fig. S15), which is also outermost-shell-specific (Experimental details), is well known to increase with the defect density^38^. Therefore, the electrochemical performance (Fig. 3) should be attributed to the corresponding defect density variations in the *outermost shell*. However, among the possible defect-generating strain sources for the outermost shell, the shell curvature should be excluded, since it *decreased* with temperature (since the particle radii increased accordingly: Figs 1 and 2). Therefore we are left with the other possible origin: the volume expansion of the diamond core on its transformation to the graphitic shells. Such strain should depend upon the extent of the phase transformation, *i.e.* the cumulative volume of the inner shells generated from the core, which dilated the outermost shell to the eventual OLC *particle size* at the end of each annealing cycles (Fig. 2). Such line of reasoning reconciled the similarity between the temperature dependence of the particle size (Fig. 2) and those of the electrochemical performance (Fig. 3) and Rct^−1^ (Fig. S18). Such conclusion was further supported, at least partly, by the similar temperature dependences of the I_D_/I_G_ ratio (Fig. 6) and the strain (Fig. 4) derived from the Raman spectral signal, although the signal originate from all the shells rather than exclusively from the outermost shell (Experimental details); it will be discussed further later. Nevertheless, some additional processes might have also contributed to the OLC particle size increase during annealing: the merging of the neighboring OLC shells as actually observed occasionally (Fig. 1d), and the Ostwald ripening^39^. The effect of these additional processes on the annealing-induced particle size evolution is not well established yet, not only in the present work but also in the previous reports on nanodiamond-derived OLC. However, these additional processes and the aforementioned core expansion are not mutually exclusive; they should proceed simultaneously, so that the actual contribution from the latter process is unquestionable.

Let us extend the analyses to the inner shells, which comprises majority of the OLC particle volume. The aforementioned forced tangential strain should occur not only at the outermost shell but also for the series of other consecutively generated shells, until the complete consumption of the diamond core. As the radial location of the newly-generated inner shells approaches particle center with gradual consumption of diamond core, their radii would decrease, and their curvatures and curvature-induced strains would increase. By contrast, since the incremental volume associated with the new-born inner shell should diminish according to their decreasing radii, the relevant incremental strain in the outer shells should accordingly diminish. Apparently, there should be gradual transition between the contributions from these two strain sources with the radial location of the new-born inner shells; they should be regarded as mutually complementary, *i.e.* as one of them increases with the radial location, the other decreases accordingly, and *vice versa*. It implied that the summed-up “strength” of these two strain sources would vary rather more weakly than their respective strengths, with the radial locations of the shells. Such expected tendency was strongly supported by the similar temperature-dependences of the properties inferred from the all-shells-encompassing Raman spectral signals (Figs 4 and 6) and the outermost-shell-specific properties (DOS, electrochemical performances, Rct^−1^: Figs 3 and 6).

Now let us estimate the core-expansion-induced strain in the outermost shell, for further discussions. Let us take OLC-1200 (Fig. 1c) as an example. From its averaged particle sizes after zero-time annealing (Fig. S7) and that after 1 hour (Figs 2 and S5c), the tangential linear strain for the *outermost* shell of OLC-1200 was estimated to be about 25% (let us refer to the large strain of such nature, hereafter, as the *total strain*). By contrast, the critical rupture strain of the graphene lattice was reported to be only 6~8% (at the temperatures of the present experimental range)^33^. Therefore the only way to accommodate the 25% strain is the ruptures of the molecular bonds, or the vacancy/void formation, which would involve the plastic deformation of the shell in addition to its elastic deformation. Such argument should be applied also for the OLC samples synthesized at different annealing temperatures. The total strain in the outermost shell should obviously depend upon the number of the generated inner shells during the annealing cycle, and hence on the annealing temperature.

However, the measured strain (Fig. 4), derived from Raman spectral signal, was one order of magnitude smaller than the aforementioned total strain of the outermost shell. It was attributed to the two possible reasons. First, the measured strain was derived from the Raman spectral signal that originated from not only from the outermost shells but also from the inner shells; the corresponding stains of the inner shells must be smaller than the outermost shell from which we calculated the total strain. Second, the Raman spectral signal originated from the molecular bonds which *survived* the bond rupture/elimination during the vacancy formation; the non-survived bonds must have been elliminated, thereby prevented from contributing to the Raman spectral signal. Such strain of the surviving bonds after the bond eliminations would be regarded as the *residual* strain (probably the *elastic* strain). Since such residual strain is a *portion* of the total strain, the two of them must share the same origin for their temperature-dependences. Since we showed that the *total* strain should increase with temperature, so must the residual strain, in agreement with actual observation (Fig. 4).

The intriguing eventual saturations in the temperature dependences of the wide variety of properties, which started at 1200 °C, need further clarification. In considering this issue, it was important that the graphite-diamond phase boundary during the phase transformation was quasi-spherical (Fig. 1), which would concentrically converge to the particle center with the annealing time, at a given temperature: for a given time, it would converge analogously with the temperature. Since such concentric convergence of phase boundary is a strong geometrical limitation, the relevant cumulative mass evolution of the generated shell would be accordingly affected. We already observed that the portion of the shells (relative to the core) in the OLC particle indeed increased with temperature (Fig. 1). Furthermore, the temperature-dependence of the particle size (Fig. 2) indeed suggested similar temperature-dependence in the portion of the generated shell mass. But, would it also follow the same intriguing temperature-dependence mentioned so far, *i.e.* the initial increase and eventual saturation at 1200 °C? The answer to this question was given by the EELS analysis of the shell/core masses as the sp^2^ and sp^3^ carbon contents (Fig. 5)^40^. The generated shell masse, *i.e.* the sp^2^ carbon contents, remarkably replicated the temperature dependence we have been observing so far: the initial increase and saturation at 1200 °C. With the discussions made so far in the preceding paragraphs as the background, such line of reasoning made in the present paragraph readily reconciled the eventual saturations in the various properties relevant to the outermost shell. It also confirmed the role of particle size (Fig. 2) as the indicator of the forced tensile strain of the outermost shell as mentioned earlier, although we did not reach the saturation issue at that time. Note that the expanding inner shells were the defect generator while the accordingly strained outer shells were defect accommodator in the preceding analyses. By contrast, for the other strain source (the curvature-induced strain), the roles of defect generator and the defect accommodator are played by the same given shell, so that the relevant analyses is rather more difficult; we put it to the future study.

**Figure 7:**
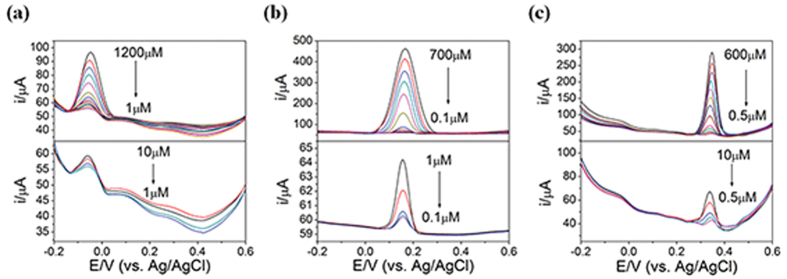
DPV responses from OLC-1200 electrode for 0.1 M PBS containing (**a**) 1–1200 μM AA, (**b**) 0.1–700 μM DA, (**c**) 0.5–600 μM UA.

Finally, for a complete assessment of the OLC’s performance in electrochemical detection, the limit of detection and the linear range of detection should be analyzed. For this purpose, the DPV responses of the OLC-1200 electrode were recorded for varying concentrations of the three analytes, in the potential range between −0.2 V to 0.6 V, as shown in Fig. 7. The DPV responses for AA, DA, and UA distinctly increased with their respective concentrations. The oxidation peak currents vs analyte concentration profiles, obtained from Fig. 7, were plotted in Fig. S19; their detection limits, linear range, and linear regression equations, obtained from Fig. S19, were summarized in Table S7. As shown in Fig. S19, the gradient of the oxidation current profiles of DA and UA underwent a transition around 60 μM and 50 μM, respectively (For discussions for the origin of such transition, see Statement S5). Also note that of AA did not undergo such transition. Such differing behaviors were attributed to the differing molecular structures of the two parties of the analytes (see Statement S5 for further comments). As shown in Table S7, the linear range of detection for AA, DA, and UA were 1~1200 μM, 0.1~700 μM, and 0.5~600 μM with the *measured* detection limit of 1000 nM, 100 nM, 500 nM and *calculated* detection limits of 760 nM, 11 nM, 36 nM, respectively. For the DA detection, the sensitivity (slope of the peak current vs concentration profile), the detection limit and the linear range were summarized in Tables S8 and S9, respectively. It was again remarkable that such performance of the optimized post-treatment-free OLC electrode, OLC-1200, was comparable to those of the n-C electrodes post-treated by nitrogen doping, catalyst metal or oxide decoration, as shown in Table S8 and S9.

Figure S20 shows the DPV response for the AA, DA, and UA detection in the presence of the interference molecules, recorded at the fixed concentrations of the two interference molecules while varying the concentrations of the target molecules. Note that the wide variations in the target molecule concentrations had negligible effect on the peak potentials and peak currents of the interference molecule; they were identical to those shown in Fig. 7 obtained in the absence of the interference molecules, which indicated the excellent selectivity of the present OLC electrodes. The measured detection limits of the present post-treatment-free OLC electrodes were 1000 nM, 250 nM, 500 nM, while their calculated detection limits were 860 nM, 30 nM, 45 nM, respectively for AA, DA, and UA, which were comparable to those of the various n-C electrodes in the previous works, which were post-treated in various ways (see Table S10). Further analyses by chrono-amperometry also yielded excellent results comparable to the post-treated carbon electrodes (see Statement S6, Figs S21, S22, and Table S11).

It was important that the novel inherent strain sources clarified in the present study eliminated the inconvenience of the post-treatments required for the conventional n-C materials in electrochemical detections of AA, DA and UA, since it enabled the performance optimization within the synthesis cycle.

## Methods

### Materials

All materials [Detonation nanodiamond powder (average size: 3.82 nm; see Fig. S1 for HR-TEM microstructure and the particle size distribution), Ascorbic acid, Dopamine hydrochloride, Uric acid, potassium ferricyanide, phosphate-buffered saline, and potassium chloride] were purchased from Sigma Aldrich Co. All aqueous solutions of the analytes were prepared with deionized water (resistivity = 18.2 MΩ/cm). The AA, DA and UA solutions were freshly prepared in 0.1 M phosphate buffer solutions (PBS, pH = 7.0) prior to use. The potassium ferricyanide solution was prepared in 1 M potassium chloride (KCl) solution.

### Synthesis of OLCs

The OLCs were synthesized by vacuum annealing of the detonation nanodiamond powder. For the annealing, 0.5 grams of nanodiamond powder was put in the graphite crucible in the vacuum furnace, equipped with the graphite heater thermally insulated by the carbon felt in a water-cooled stainless-steel vacuum chamber, evacuated with rotary pump to 10^−3^ Torr. The annealing temperature was varied as 1000 °C, 1100 °C, 1200 °C, and 1400 °C; the annealing time was fixed at 1 hour. The synthesized OLC samples were accordingly denoted as OLC-1000, OLC-1100, OLC-1200, and OLC-1400, respectively. For comparison, an additional sample was made by the same annealing process at 1200 °C with zero annealing time. The temperature was elevated at a rate of 10 °C/min. at the ramp stage. The samples were furnace-cooled to room temperature after the annealing.

### Structural Characterization of OLC

The microstructure and crystalline structure of the OLC were characterized by high-resolution transmission electron microscopy (HR-TEM, FEI Co, Titan 300 kV), electron energy loss spectroscopy (EELS, FEI Co, Titan 80 kV, penetration depth^41^: about 20~30 μm) and x-ray diffraction (XRD, Rigaku Co, D/Max 2500 V). The strain and defect density were analyzed by the visible-Raman spectroscopy (Renishaw Co., inVia Raman spectroscopy, The spectral resolution: 0.5 cm^−1^), employing the 532 nm Nd:YAG laser; its penetration depth was 10–30 nm^42^, one order of magnitude larger than the OLC particle diameter. Since the visible-Raman is 50~230 times more sensitive to sp^2^-carbon than sp^3^-carbon, the signal arising from the sp^3^ carbon was negligible^43^. The density of states (DOS) was characterized by the ultraviolet photoemission spectroscopy (UPS, Ulvac Co., PHI 5000 Versaprobe), employing a He(I) emission lamp (photon energy: 21.2 eV); the signal collection was made with a 0.01 eV resolution. The electron take-off angle was 90°; the pass energy was 0.585 eV. Gold was used as the reference sample. The electron escape depth (or electron mean free path) of the UPS spectra was estimated to be a few Å (section 1.2 of the reference) for the employed photon energy of 21.2 eV^44^; the UPS signal should have originated largely from the outermost shell.

### Preparation of working electrodes

Prior to the OLC drop-coating, the glassy carbon electrode (GCE) of 3 mm in diameter was polished successively with 1 μm and 0.05 μm alumina abrasive powders. It was subsequently rinsed twice with aqueous solution of Isopropyl alcohol for 5 min, respectively, and was dried by blowing nitrogen gas. Two milligrams of synthesized OLC powder were mixed with isopropyl alcohol (0.5 mL) and deionized water (0.5 mL), and was subsequently subjected to the ultrasonic agitation. A 5 μL droplet of the suspension was dropped onto the pre-treated GCE surface and was dried at room temperature to form a drop-coated layer of the OLC powder.

### Electrochemical measurement

All of the electrochemical measurements were performed with a VSP Potentiostat/Galvanostat (Bio-logic Co., France). A conventional three-electrode system was used throughout the measurements. The glassy carbon electrode, modified by the drop-coated OLC powder, was employed as the working electrode. The DPV performance of bare glassy carbon electrode was negligible compared to the OLC drop-coated electrode as shown in Fig. S23. A platinum wire and an Ag/AgCl electrode were used as the counter electrode and the reference electrode, respectively. The experiments were carried out at room temperature. The electrochemical impedance spectroscopy was carried out at 0.3 V from 200 kHz down to 0.1 Hz with 10 mV AC amplitude. The Warburg impedance was analyzed employing the rotating electrode technique with the rotation speed of 500~2500 rpm^45^. All electrochemical impedance measurements were carried out in a 1 M KCl solution containing 1 mM ferricyanide.

## Additional Information

**How to cite this article**: Ko, Y.-J. *et al.* Inherently-Forced Tensile Strain in Nanodiamond-Derived Onion-like Carbon: Consequences in Defect-Induced Electrochemical Activation. *Sci. Rep.* **6**, 23913; doi: 10.1038/srep23913 (2016).

## Supplementary Material

Supplementary Information

## Figures and Tables

**Table 1:** Summary of the oxidation peak currents and potentials for the detection of AA, DA and UA obtained by CV and DPV employing the OLC electrodes.

Analyte	Electrode material	CV: Individual detection (1 mM)		CV: Simultanous detection (0.33 mM, respectively)		DPV: Simultanous detection (0.33 mM, respectively)	
		I_P_(μA)	E_P_(V)	I_P_(μA)	E_P_(V)	I_P_(μA)	E_P_(V)
AA	OLC-1000	5	0.110	2	0.110	0.3	0.024
	OLC-1100	30	−0.018	10	0.019	0.5	−0.011
	OLC-1200	41	−0.055	15	−0.032	20	−0.054
	OLC-1400	42	−0.053	15	−0.015	21	−0.047
DA	OLC-1000	44	0.275	15	0.286	10	0.165
	OLC-1100	55	0.219	20	0.212	30	0.170
	OLC-1200	112	0.210	38	0.207	220.5	0.169
	OLC-1400	109	0.209	37	0.208	217.5	0.173
UA	OLC-1000	32	0.410	12	0.412	12	0.35
	OLC-1100	43	0.355	20	0.362	22	0.319
	OLC-1200	143	0.346	50	0.349	197.5	0.331
	OLC-1400	143	0.355	53	0.352	195.1	0.335
