# Supplementary material for: Inherently-Forced Tensile Strain in Nanodiamond-Derived Onion-like Carbon: Consequences in Defect-Induced Electrochemical Activation
Source: Sci Rep. 2016 Apr 1;6:23913. doi: 10.1038/srep23913 (PMC4817152; doi:10.1038/srep23913)
Supplement: Supplementary Information [file srep23913-s1.doc]

**Supplementary Information**

**Inherently-Forced Tensile Strain in Nanodiamond-Derived Onion-like Carbon: Consequences in Defect-Induced Electrochemical Activation**

Young-Jin Ko,^a,b^ Jung-Min Cho,^a,b^ Inho Kim,^a^ Doo Seok Jeong,^a^ Kyeong-Seok Lee,^a^ Jong-Keuk Park,^a^ Young-Joon Baik,^a^ Heon-Jin Choi,^b^ Seung-Cheol Lee^c^ and Wook-Seong Lee^*a^

**Table S1.** The annealing-induced defect generation or healing in various nano-carbon materials in the previous reports

| Material | Temperature | Atmosphere | Healed/Generated | Reference |
| --- | --- | --- | --- | --- |
| Graphene | 100~500^o^C | Vacuum | Healed | 1 |
| Graphene | 1600~2850 ^o^C | Argon | Healed | 2 |
| Graphene nanoribbon | 1000~2800 ^o^C | Vacuum | Healed | 3 |
| MWCNT | 1600~3000 ^o^C | Nitrogen | Healed | 4 |
| SWCNT | 900 ^o^C | Vacuum | Healed | 5 |
| Onion-like carbon | 1500~2200 ^o^C | Vacuum | Healed | 6 |
| Onion-like carbon | 827~1827 ^o^C | Vacuum | Not mentioned | 7 |
| Onion-like carbon | 25~1000 ^o^C | Argon | Generated | 8 |
| Onion-like carbon | 1300~1800 ^o^C | Vacuum | Healed | 9 |
| Onion-like carbon | 550~1300 ^o^C | Helium | Generated (Up to 1100 ^o^C)  Healed (Over 1100 ^o^C) | 10 |
| Onion-like carbon | 0~2000 ^o^C | Vacuum | Healed | 11 |

**Reference**

[1] Z.H. Ni, H.M.Wang, Y.Ma, J.Kasim, Y.H.Wu, Z. X. Shen, ACS nano, 2008, 2, 1033-1039.

[2] G. Xin, H. Sun, S. M. Scott, T. Yao, F. Lu, D. Shao, T. Hu, G. Wang, G. Ran, J. Lian, ACS Applied Materials & Interfaces, 2014, 6, 15262-15271.

[3] J. Campos-Delgado, Y.A. Kim, T. Hayashi, A. Morelos-Gómez, M. Hofmann, H. Muramatsu, M. Endo, H. Terrones, R.D. Shull, M.S. Dresselhaus, M. Terrones, Chemical Physics Letters, 2009, 469, 177-182.

[4] R. Andrews, D. Jacques, D. Qian, E.C. Dickey, Carbon, 2001, 39, 1681-1687.

[5] V. Georgakilas, D. Voulgaris, E. Vazquez, M. Prato, D. M. Guidi, A. Kukovecz, H. Kuzmany, Journal of the American Chemical Society, 2002, 124, 14318-14319.

[6] S. Tomita, T. Sakurai, H. Ohta, M. Fujii, S. Hayashi, The journal of Chemical Physics, 2001, 114, 7477-7482.

[7] E. D. Obraztsova, M. Fujii, S. Hayashi, V. L. Kuznetsov, U. V. Butenko, A. L. Chuvilin, Carbon, 1998, 36, 821-826

[8] J. Cebik, J. K. McDonough, F. Peerally, R. Medrano, I. Neitzel, Y. Gogotsi, S. Osswald, Nanotechnology, 2013, 24, 205703

[9] J. K. McDonough, A. I. Frolov, V. presser, J. Niu, C. H. Miller, T. Ubieto, M. V. Fedorov, Y. Gogotsi, Carbon, 2012, 50, 3298-3309

[10] R. Wang, X. Sun, B. Zhang, X. Sun, D. Su, Chemistry – A European journal, 2014, 20, 6324-6331

[11] C. Portet, G. Yushin, Y. Gogotsi, Carbon, 2007, 45, 2511-2518


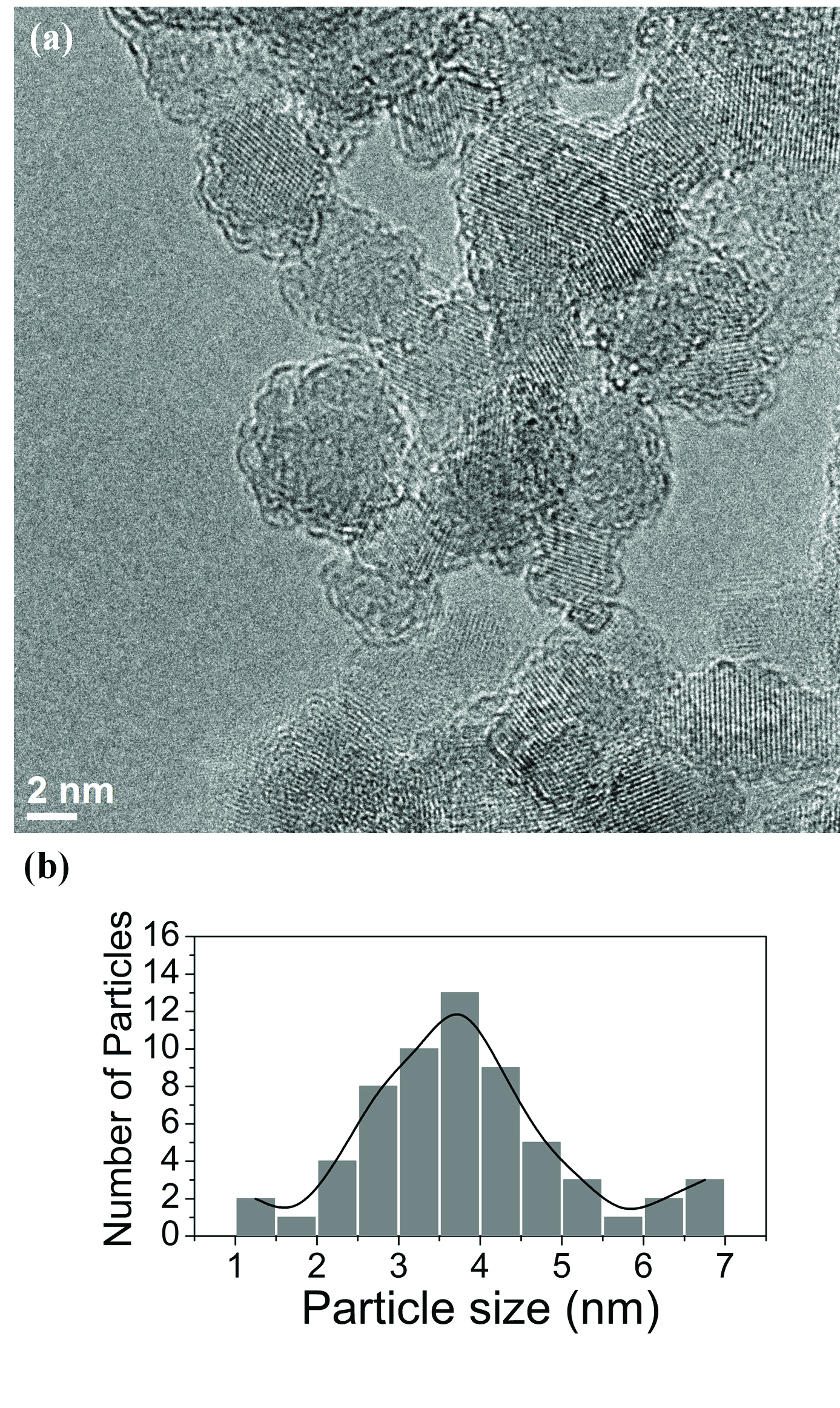


Figure S1. (a) HR-TEM image and (b) The particle size-distribution of the as-received detonation nanodiamond powder, which was obtained in the same way stated in the caption of Figure 2

**Table S2.** The inter-shell spacings of the OLC-1400 (counted from the surface) measured by the radial line scan shown in Figure S2

|  | 1st-2nd  shell | 4th-5th  shells | 7th-8th  shells | 9th-10th  shells | 10th-11th  shells |
| --- | --- | --- | --- | --- | --- |
| (b) Interplanar spacing (nm) | 0.346 | 0.324 | 0.311 | 0.296 | 0.288 |
| (d) Interplanar spacing (nm) | 0.347 | 0.312 | 0.296 | - | - |


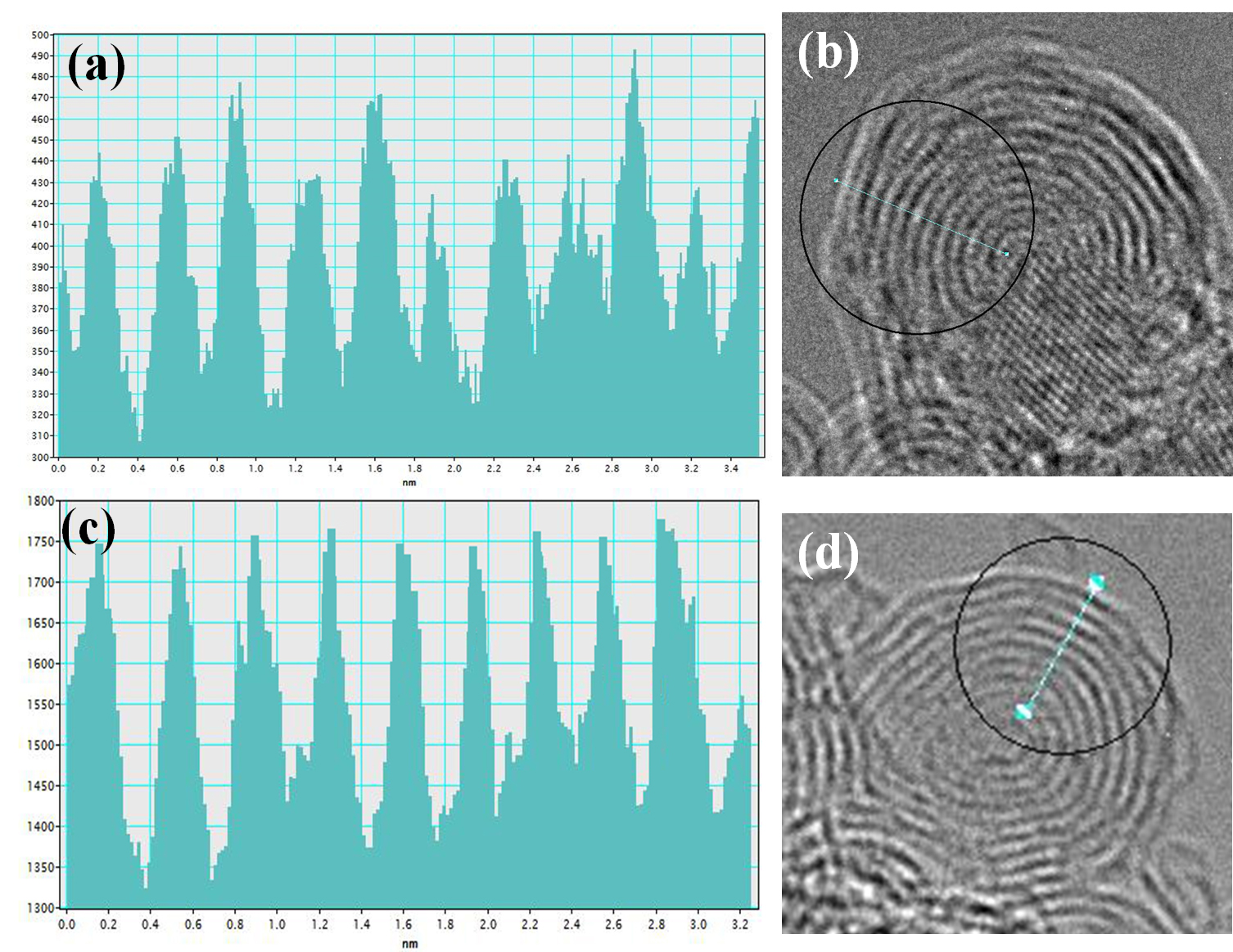


**Figure S2.** (a, c) The radial line scan profiles across the shells in the (b, d) HR-TEM images of an OLC particle, from which the Table S2 was obtained


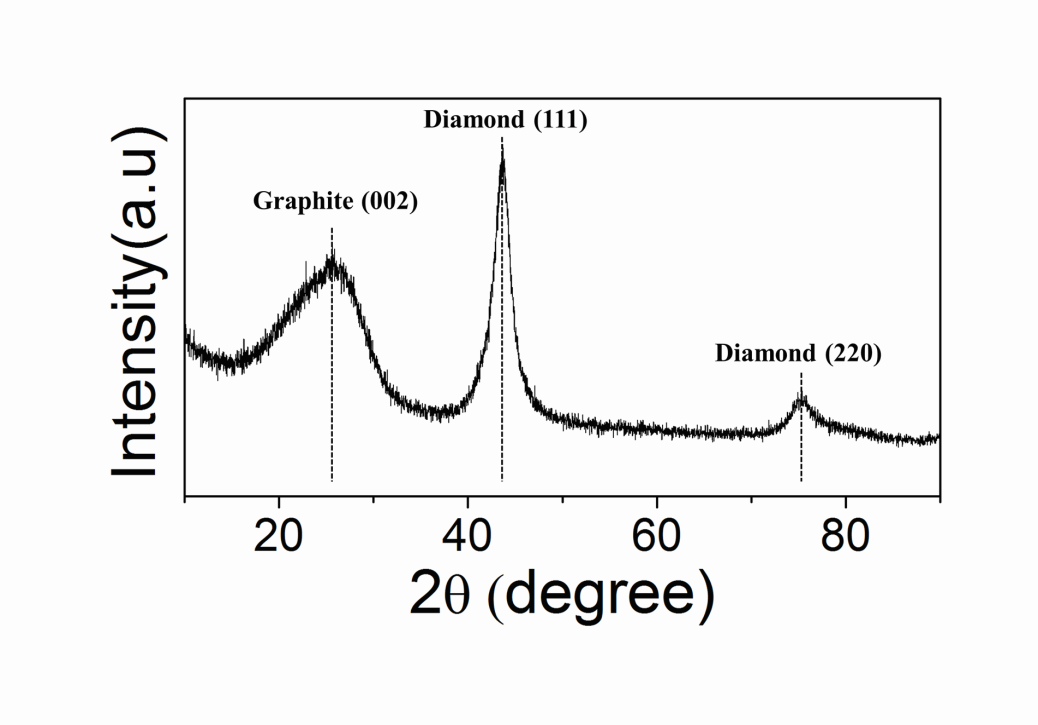


**Figure S3.** XRD pattern of the OLC nanoparticles which was synthesized at 1200°C (OLC-1200)


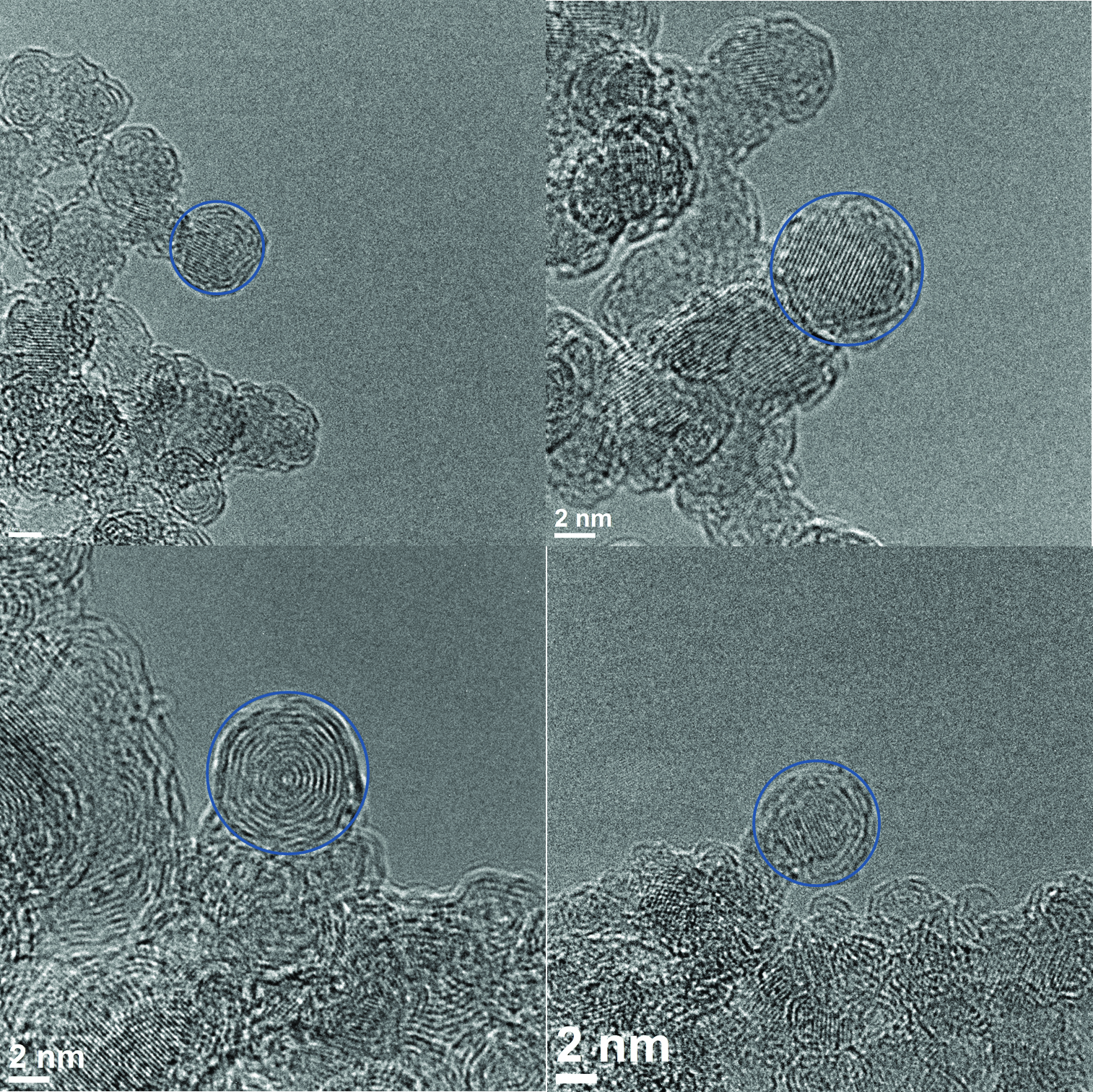


Figure S4. The examples of the HR-TEM images of isolated OLC particles (indicated by the blue solid circle) employed for obtaining the particle size distribution

**Statement S1**

Recall that the graphite-diamond core-shell nano particles (shown in Figure 1a~c) are usually called buckydiamond,^1^ while the particles without the diamond core (shown in Figure 1d) are called onion-like carbon.^1^ But partly for convenience, and partly since it was mainly the onion-like carbon shell of the particles that was important for our discussions given below, hereafter we will refer the particles simply with the name OLC.

**Reference**

[1] A.Krueger, D.Lang, Advanced Functional Materials. 2012, 22, 890–906


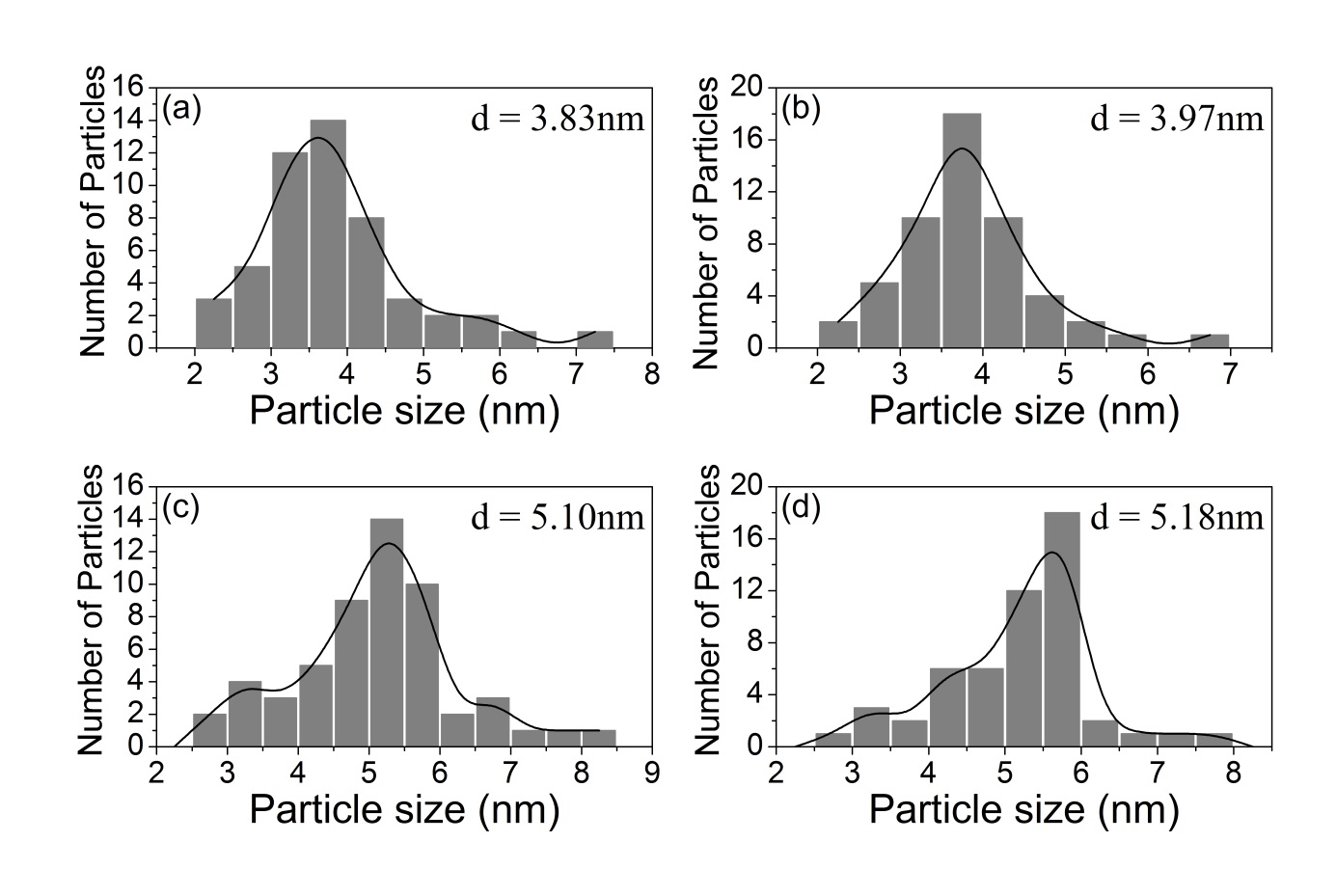


**Figure S5.** The particle size-distributions of (a) OLC-1000, (b) OLC-1100, (c) OLC-1200 and (d) OLC-1400


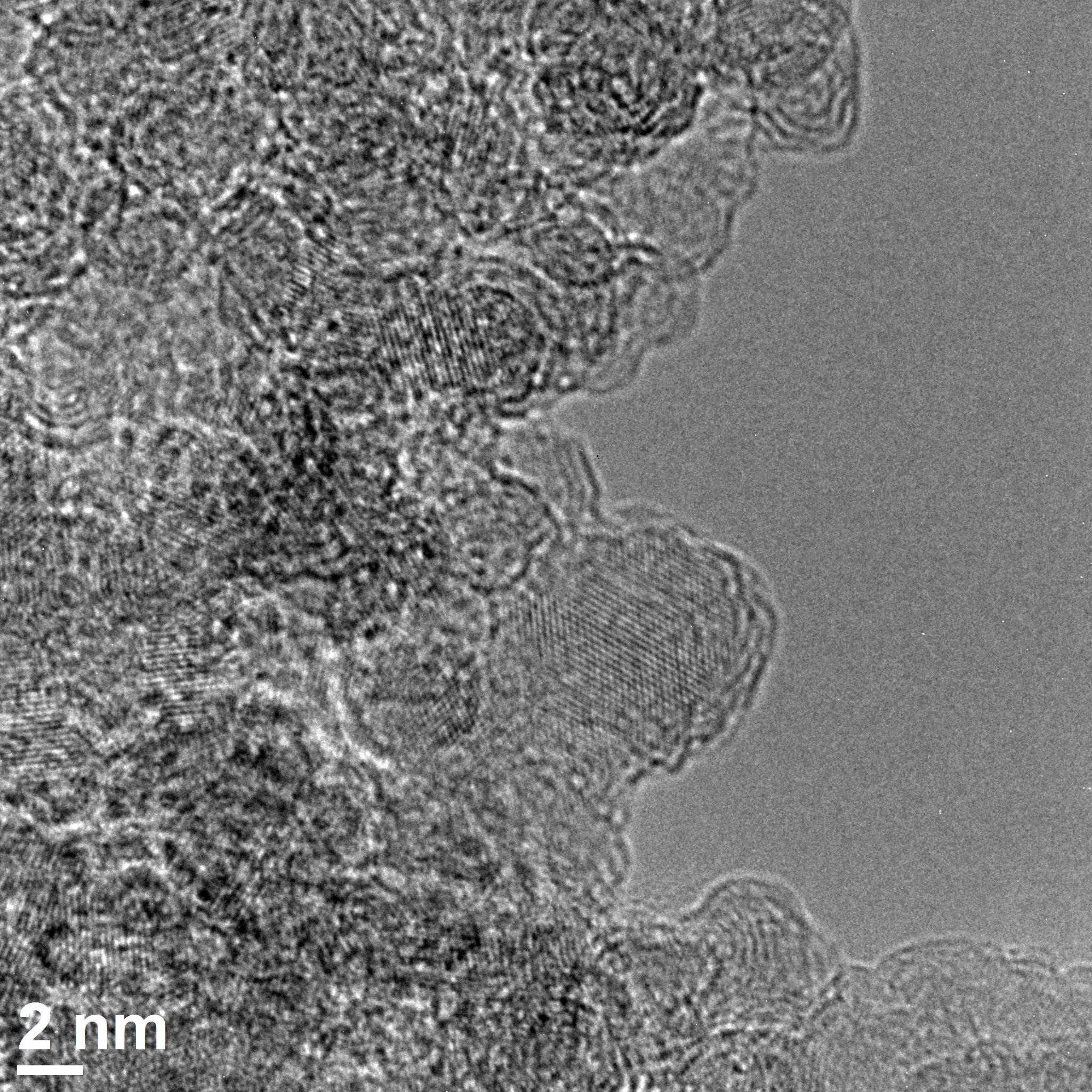


**Figure S6.** HR-TEM image of the OLC-1200 after zero-time annealing


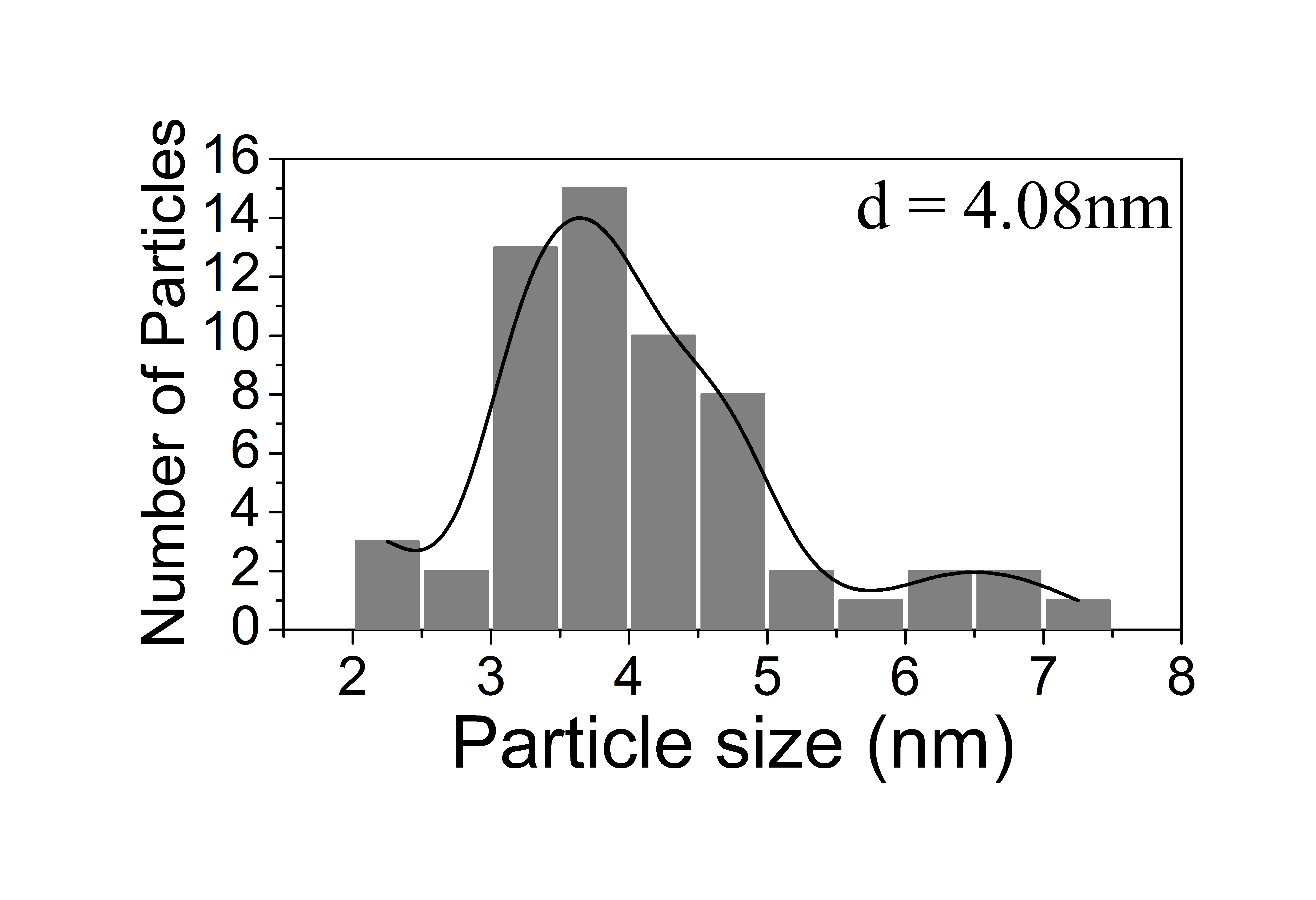


**Figure S7.** The particle size-distribution of the OLC-1200 after zero-time annealing


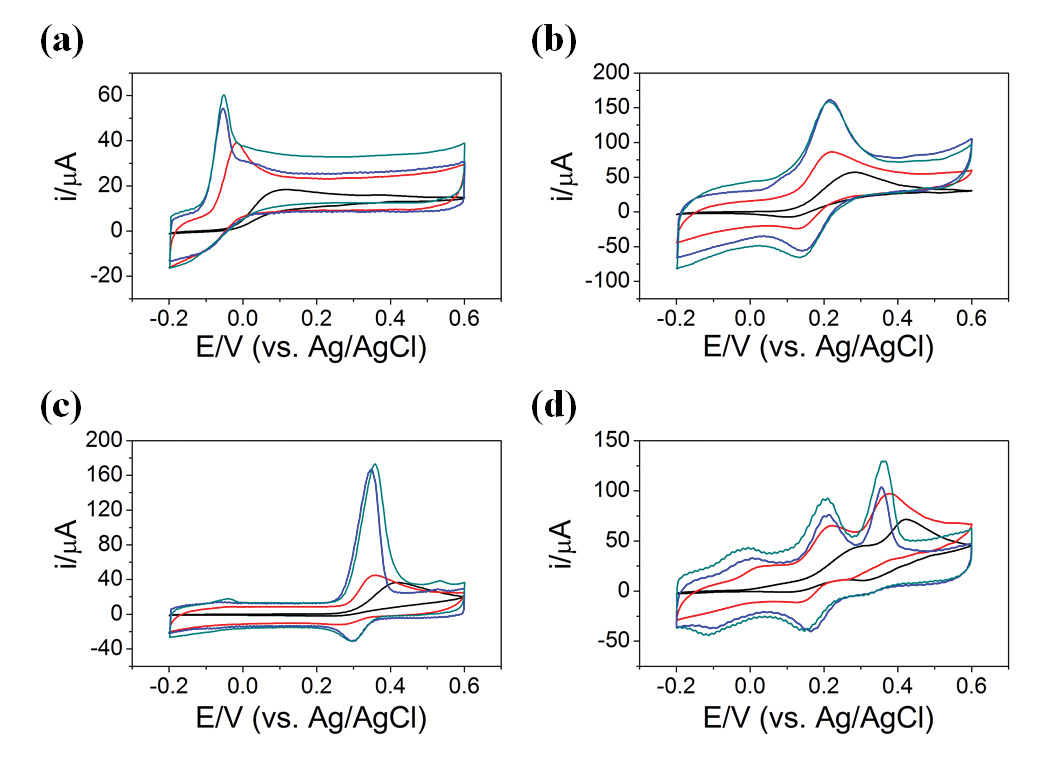


**Figure S8.** (a)~(c) CV responses from the OLC electrodes (black: OLC-1000, red: OLC-1100, blue: OLC-1200, dark cyan: OLC-1400) for the 0.1M PBS respectively containing 1mM of (a) AA, (b) DA, (c) UA, and (d) 0.33mM AA + 0.33mM DA + 0.33mM UA.


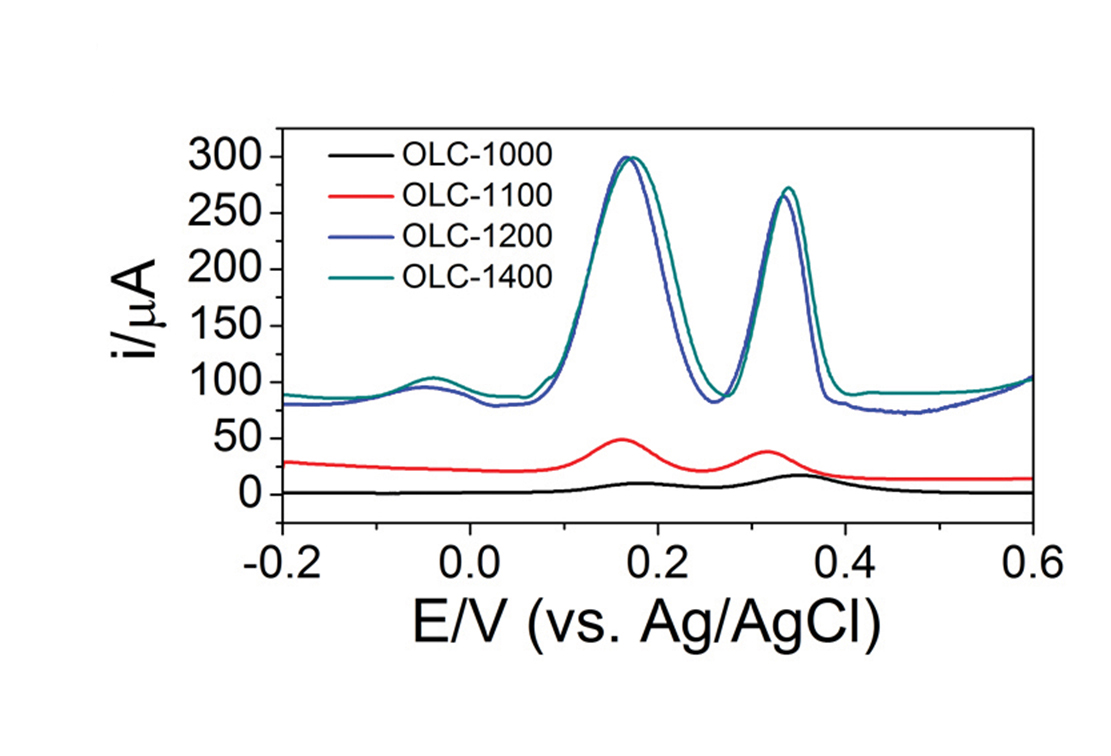


**Figure S9.** (a) DPV responses from the OLC electrodes for the mixture solution containing 0.33mM AA + 0.33mM DA + 0.33mM UA

**Table S3**. Summary of the previous reports concerning the oxidation peak separations in the simultaneous detection of AA, DA and UA obtained by DPV, as compared to those in the present study

| Electrode material | ΔE_(DA-AA)_ | ΔE_(UA-DA)_ | ΔE_(UA-AA)_ | Ref |
| --- | --- | --- | --- | --- |
| Polystyrene sulfonate wrapped multiwall-CNT | 210mV | 119mV | 329mV | 1 |
| N-graphene | 200mV | 132mV | 332mV | 2 |
| GONR | 229.9mV | 126.7mV | 356.6mV | 3 |
| Pt/Graphene | 240mV | 120mV | 360mV | 4 |
| Graphene-SnO2 | 132mV | 128mV | 260mV | 5 |
| N-doped porous carbon nanopolyhedra | 228mV | 124mV | 352mV | 6 |
| OLC-1200 | 223mV | 162mV | 385mV | This work |

**Reference**

[1] R.Manjunathaa, G.S.Suresha, J.S.Melob, S.F.D’Souzab, T.V.Venkateshac, Sensors and Actuators B, 2010, 145, 643-650.

[2] Z.H.Shenga, X.Q.Zhenga, J.Y.Xua, W.J.Baoa, F.B.Wanga, X.H.Xiaa, Biosensors and Bioelectronics, 2012, 34, 125-131.

[3] C.L.Sun, C.T.Chang, H.H.Lee, J.Zhou, J.Wang, T.K.Sham, W.F.Pong, ACS nano, 2011, 5, 7788-7795.

[4] C.L.Suna, H.H.Leea, J.M.Yanga, C.C.Wub, Biosensors and Bioelectronics, 2011, 26, 3450-3455.

[5] A.Yang, Y.Xue, Y.Zhang, X.Zhang, h.Zhao, X.Li, Y.He, Z.Yuan, Journal of materials chemistry B, 2013,1, 1804-1811

[6] P.Gai, H.Zhang, Y.Zhang, W.Liu, G.Zhu, X.Zhang, J.Chen, Journal of materials chemistry B, 2013, 1, 2742-2749

**Statement S2**

The oxidation potential of the analyte is built up by two different Schottky barriers (1) between the conduction band edge of the working electrode and that of the LUMO of the analyte; (2) between the counter electrode (Pt) and the HOMO of the analyte. The net oxidation potential is given as E (Pt) – Ec (working electrode) + LUMO (analyte) – HOMO (analyte).^1^ Therefore, when the working electrode and the counter electrode is given, the net oxidation potential is exclusively determined by the LUMO-HOMO gap of the analyte molecule; the differing values of such gaps of the analyte molecules serve as the prime source of the peak separation; it provides selectivity for the electrochemical detection. When the analyte molecules are confined to the electrode surface (by adsorption, for example), it induces the dipole interaction with the electrode surface, which further modifies the oxidation potential.^1^ On the other hand, when the analytes and counter electrode are given as in the present study, the net oxidation potentials of the analyte are exclusively determined by the conduction band edges of the (Ec) of the working electrode material. In Table S3, the target analytes were fixed as AA, DA, and UA, while the working electrode materials were varied. The relatively small change of the peak separations among the various electrodes strongly suggested the accordingly small variation among the conduction band edges (Ec) of the various electrode materials listed in the Table S3.

**Reference**

[1] H. Y. Yue, S. Huang, J. Chang, C. Heo, F. Yao, S. Adhikari, F. Gunes, L. C. Liu, T. H. Lee, E. S. Oh, B. Li, J. J. Zhang, T. Q. Huy, N. V. Luan, Y. H. Lee, Acs Nano, 2014, 8, 1639-1646.

**
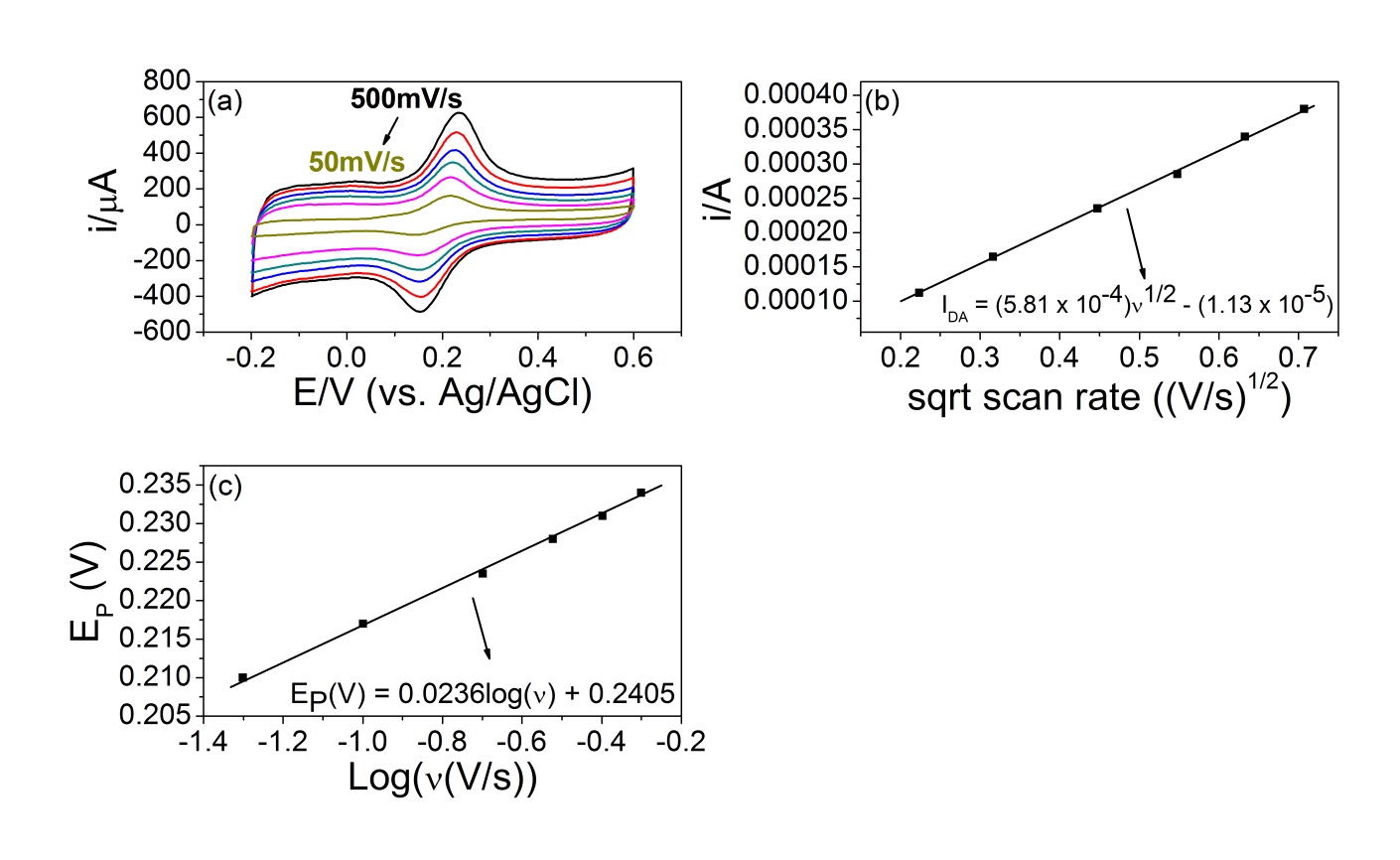
**

**Figure S10.** (a) CV responses from the OLC-1200 electrode for 1mM DA at the various scan rates (50~500mV/s); corresponding plots of (b) the oxidation peak current vs square root scan rate and (c) the oxidation peak potential vs logarithm of the scan rate.

**
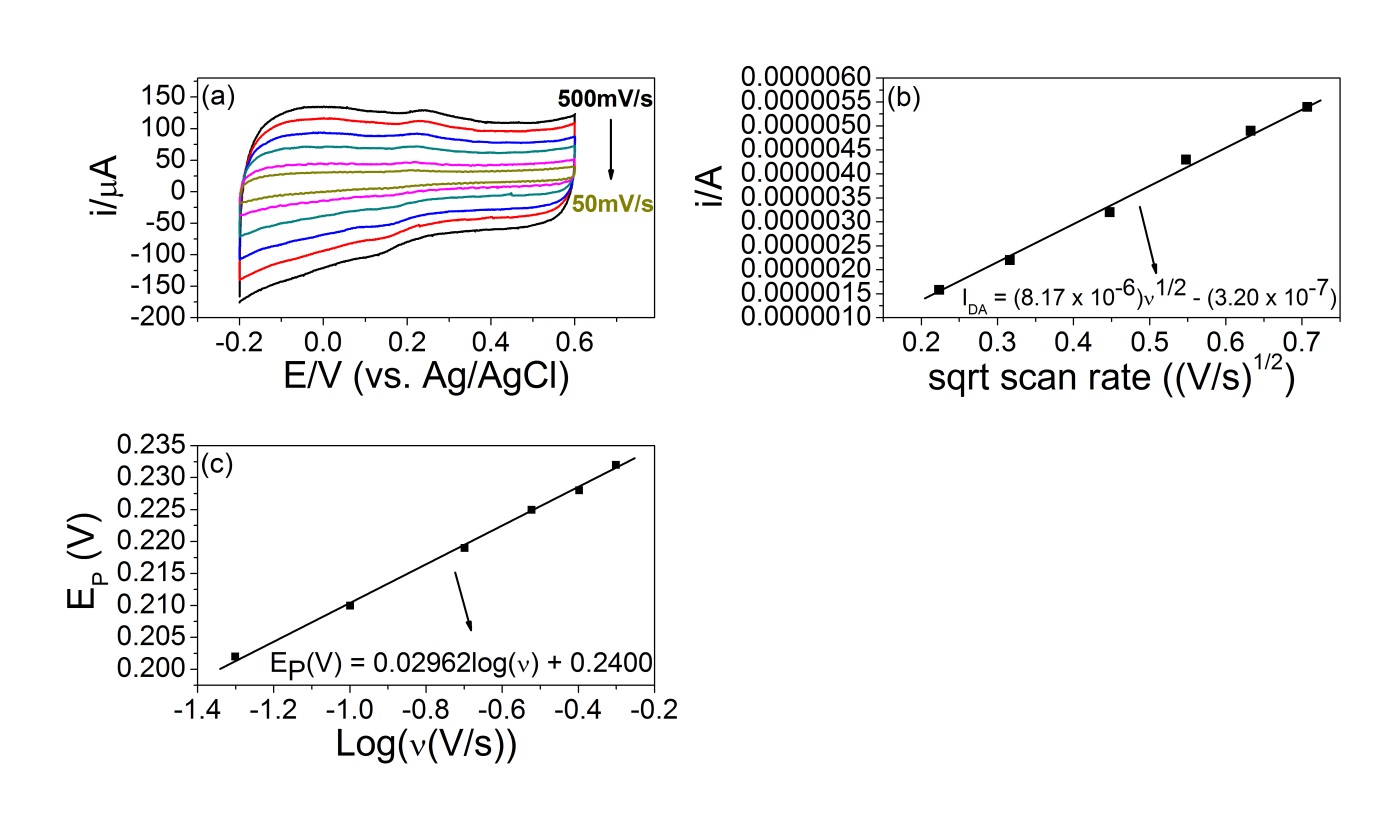
**

**Figure S11.** (a) CV responses from the OLC-1200 electrode for 1μM DA at the various scan rates (50~500mV/s); the corresponding plots of (b) the oxidation peak current vs square root scan rate and (c) the oxidation peak potential vs logarithm of the scan rate.

**Statement S3**

To analyze the rate-determining steps of the electrochemical oxidation reaction on the present OLC electrode, the peak current and the peak positions in the CV responses from the OLC-1200 were plotted against the square root scan rate for 1mM and 1μM DA, respectively (See Figure S10, S11). The oxidation peak current showed linear dependence on the square root of scan rate, which indicated that the DA oxidation was controlled, at least partly, by the diffusion.^1^ We have also plotted the oxidation peak position against the logarithm of scan rate, to test the possibility of the electron-transfer-control.^1^ The plot was also linear, which indicated the DA oxidation reaction was also controlled by the electron-transfer. It indicated that the DA oxidation reaction was mixed-controlled.^1, 2^

**Reference**

[1] H. Razmi, A. Azadbakht, Electrochimica Acta, 2005, 50, 2193-2201.

[2] Z. Conzalez, C. Botas, P. Alvarez, S. Roldan, C. Blanco, R. Santamaria, M. Granda, R. Menendez, Carbon, 2012, 50, 828-832.


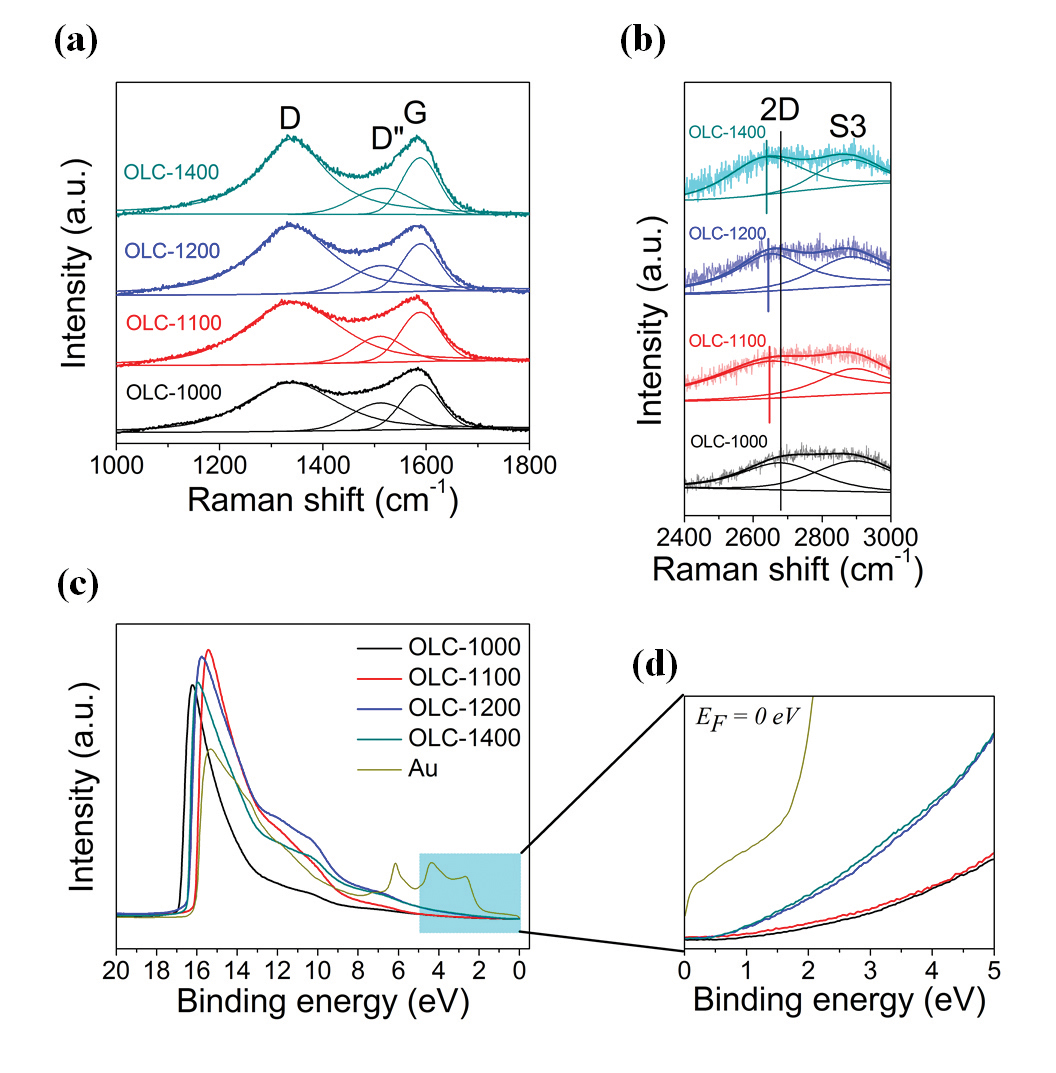


**Figure S12.** (a-b) Raman spectra [(a): D and G band; (b): 2D band] and (c-d) UPS spectra of OLCs synthesized at various annealing temperatures [(d): blow-up view of the marked area in (c)].


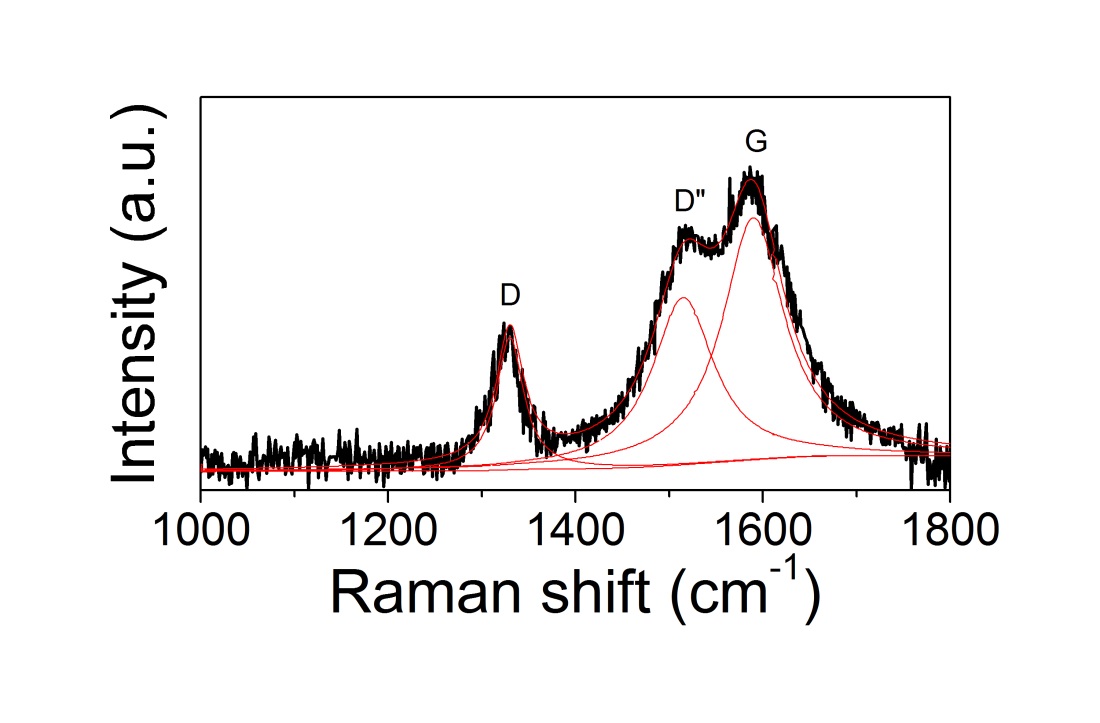


**Figure S13.** The back-ground substracted Raman spectrum obtained for the as-received detonation diamond power adopted as raw material in our experiment. The I_D_/I_G_ ratio obtained from the spectrum was only 0.48, which was only 46% of that of OLC-1000 (of which the I_D_/I_G_ ratio was the lowest among our OLC samples). It indicated the negligible, if any, contribution from the initial defects in the initial raw nanodiamond powder to the I_D_/I_G_ ratio in the Raman spectra of the OLC samples.

**Table S4**. Summary of the peak frequencies of D band (V_D_), G band (V_G_), 2D band (V_D″_), and the I_D_/I_G_ ratio in the Raman spectra of the OLCs synthesized at various annealing temperatures

| Sample | V_D_  (cm^-1^) | V_G_  (cm^-1^) | V _2D_  (cm^-1^) | I_D_/I_G_ |
| --- | --- | --- | --- | --- |
| Detonation  nanodiamond | 1334 | 1590 | - | 0.48 |
| OLC-1000 | 1335 | 1586 | 2676 | 1.03 |
| OLC-1100 | 1342 | 1589 | 2658 | 1.15 |
| OLC-1200 | 1340 | 1589 | 2643 | 1.40 |
| OLC-1400 | 1340 | 1590 | 2635 | 1.41 |

* Peak deconvolution was carried out according to the methods reported in the references.^1^

**Reference**

[1] C. Kim, S. H. Park, J. K. Cho, D. Y. Lee, T. J. Park, W. J. Lee, K. S. Yang, Journal of Raman Spectroscopy, 2004, 35, 928-933; V.C.Tung, M.J.Allen, Y.Yang, R.B.Kaner, Nature Nanotechnology, 2009, 4, 25-29.


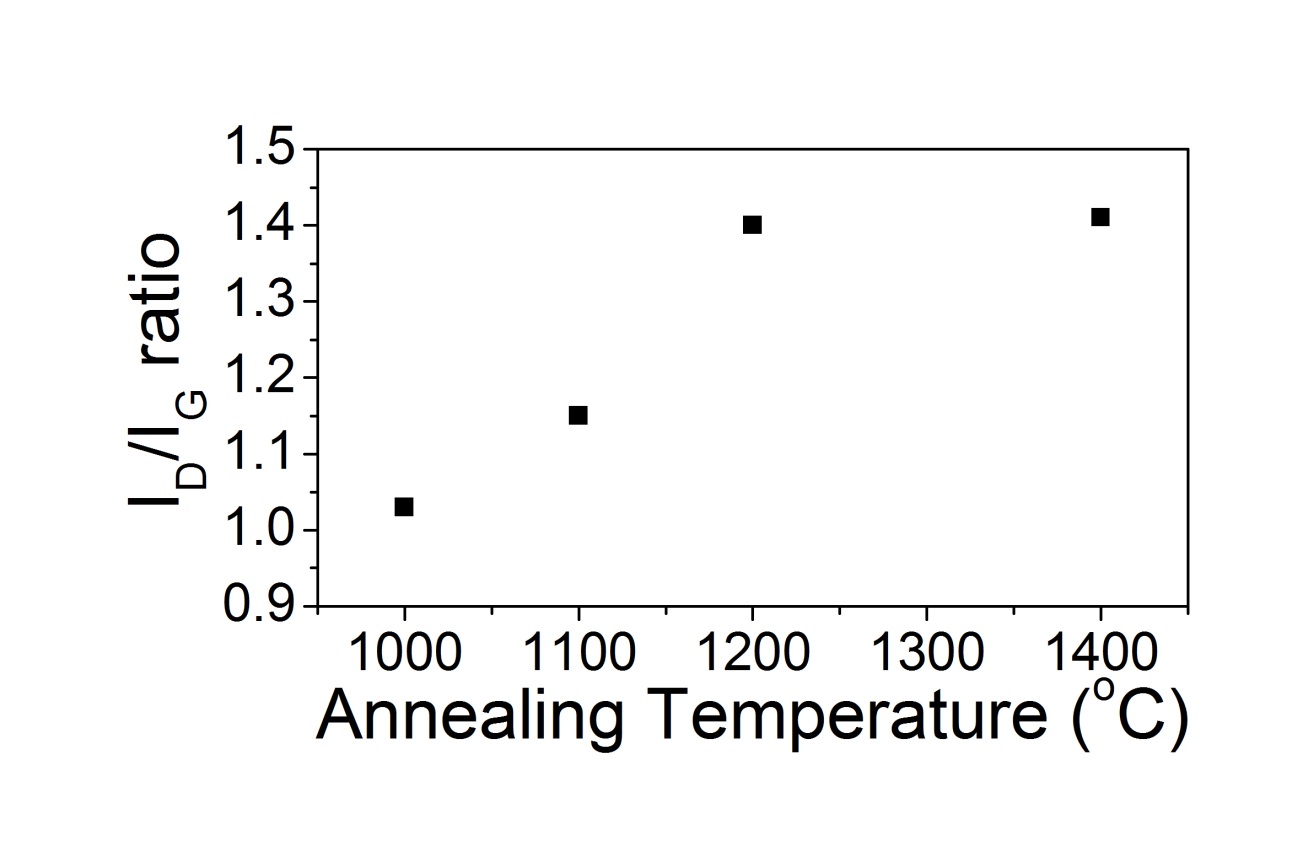


**Figure S14**. The I_D_/I_G_ ratio *vs* annealing temperature profile corresponding to Table S4


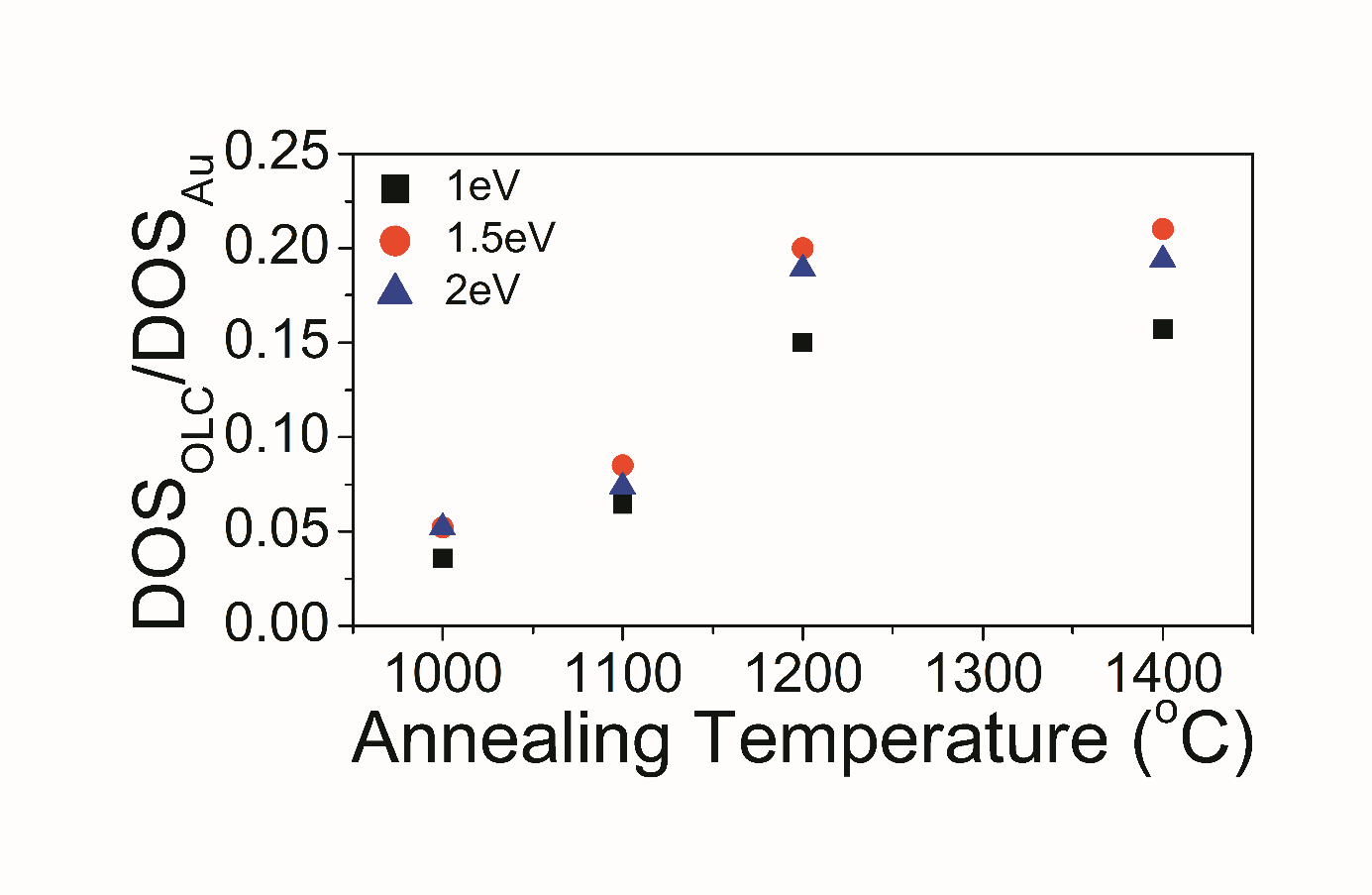


**Figure S15.** The DOS of OLCs (normalized by the DOS of Au) *vs* annealing temperature profiles at various binding energy, corresponding to Figure S12c, d.

**Statement S4**

The Nyquist plot^1^ obtained from the impedance spectroscopy employing the ordinary non-rotating working electrode was given in Figure S15. Each curve in the Figure S16 consisted of a partial semi-circle at the high-frequency domain, and the linear portion extending therefrom toward the low frequency domain. For the lower annealing temperatures (OLC-1000, OLC-1100), the existence of the semi-circles was rather obscure, while it was increasingly clarified with annealing temperature (OLC-1200 and OLC-1400). Such a characteristic curve shape was possibly attributed to the typical electrode reactions under the mixed-control by (1) the electron-transfer through the electrode/solution interface and (2) the diffusion of the analyte through the solution (section 5.2.2.3 of reference).^1^ The semi-circle at the high-frequency domain represents the impedance against the electron-transfer through the electrode/solution interface, while the linear portion represents the impedance in the Warburg region, *i.e.*, that of the analyte diffusion in the solution (section 5.2.2.5 of the reference).^1^ Such a possibility can, in general, be confirmed by employing the rotating disk electrode technique, where the working electrode is rotated in the solution.^1^ It induces the forced convection around the electrode which causes the linear portion of the plot to curve back toward the horizontal axis to form the second semi-circle; the radius of the semicircle decreases with the rotation speed.^1^

Figure S17 shows the Nyquist plot obtained by employing the rotating disk technique, which clearly demonstrated that the linear portion indeed curved back to form the 2nd semi-circle, whose radii decreased with rotation speed. The semi-circles for OLC-1200 and OLC-1400 were discernible only in the small-scale plot shown in Figure S17e, f. By contrast, the semi-circles for OLC-1000 and OLC-1100 were discernible not only in Figure S16a but also in Figures S17a, b, probably due to the larger impedance of them. Employing the impedance spectroscopy data presented in Figure S16 and Figure S17, the charge transfer resistance (Rct) was calculated from the radii of the first semi-circles, assuming the Randle circuit^1^ (inset of Figure S16a), as shown in Table S5 and Figure S17.

**Reference**

[1] C. H. Hamann, A. Hamnett, W. Vielstich, Electrochemistry, 2007


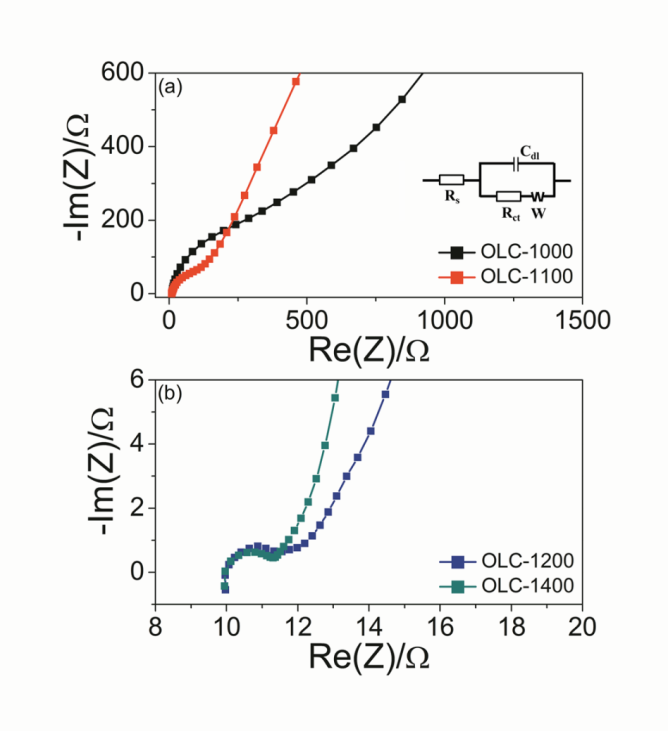


**Figure S16.** Electrochemical impedance spectra of OLC electrodes obtained from the 1M KCl solution containing 1mM [Fe(CN)_6_]^3-/4-^.


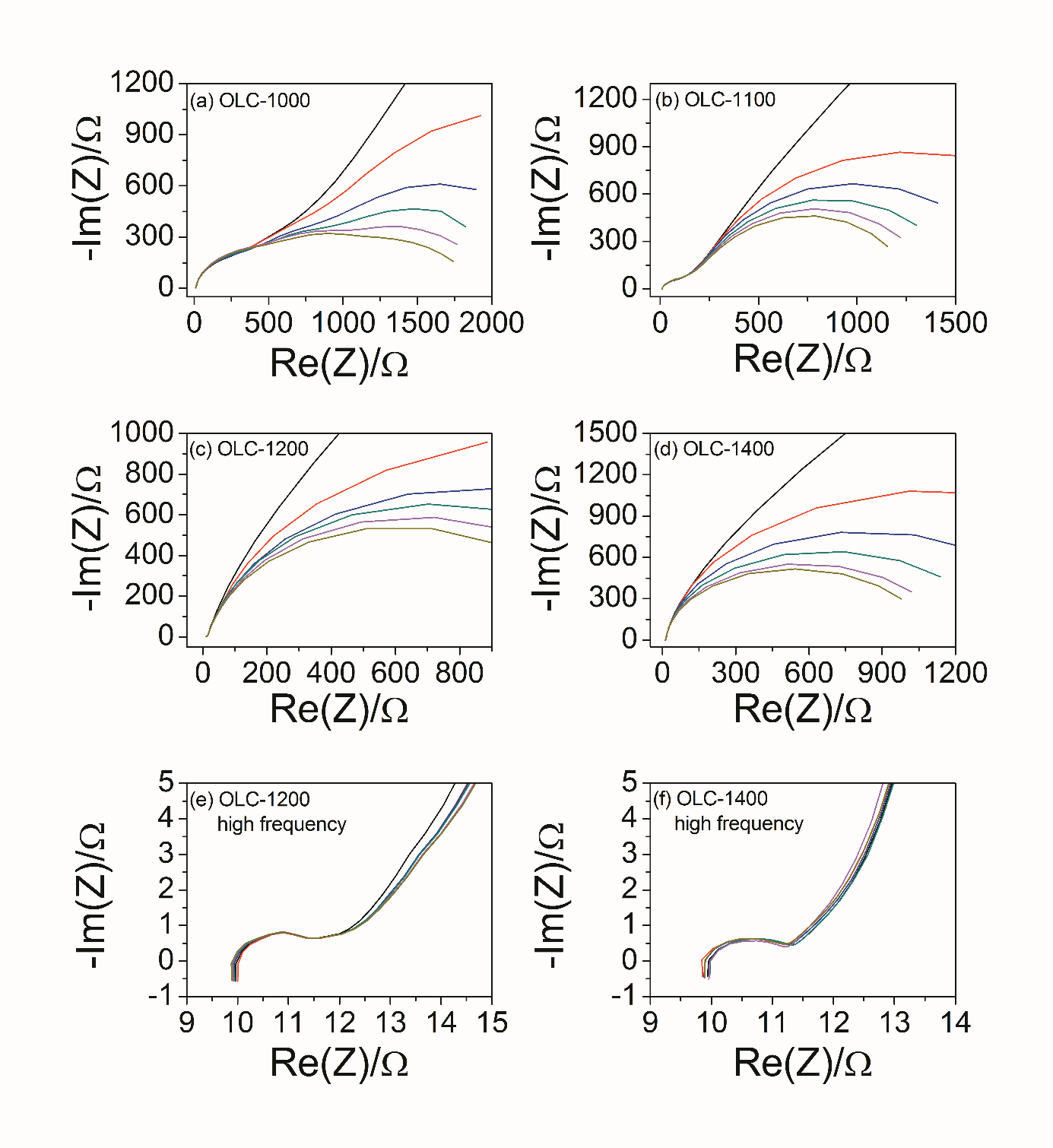


**Figure S17.** Electrochemical impedance spectra of OLC electrodes recorded using the rotating disk electrode at various rotation speeds (black: 0rpm, red: 500rpm, blue: 1000rpm, dark cyan: 1500rpm, magenta: 2000rpm, yellow: 2500rpm)

**Table S5**. Summary of Rct values obtained by electrochemical impedance spectroscopy from the OLC electrodes

| Sample | Rct  (Ω) |
| --- | --- |
| OLC-1000 | 602 |
| OLC-1100 | 249 |
| OLC-1200 | 2.12 |
| OLC-1400 | 1.96 |


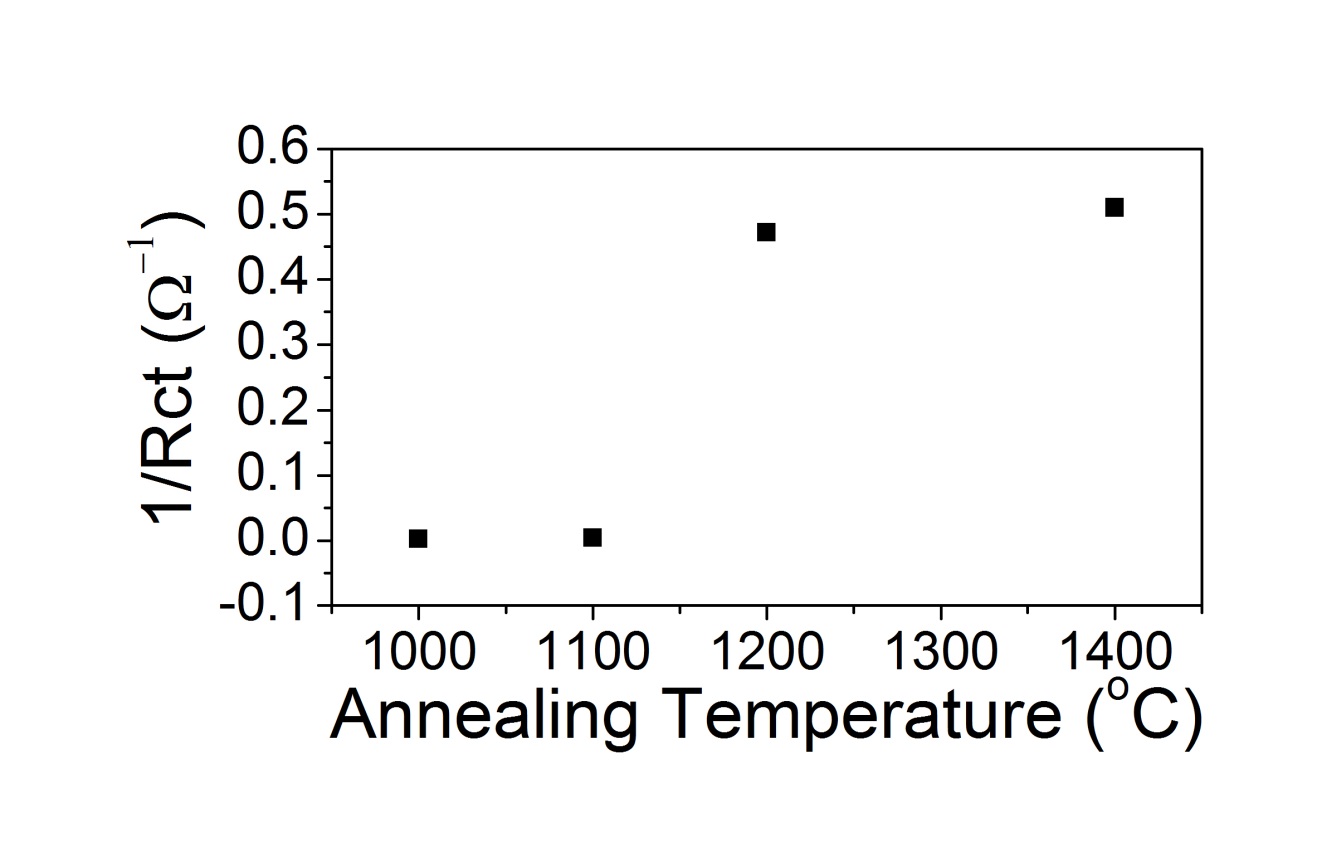


**Figure S18.** The Rct^-1^ vs annealing temperature profile corresponding to Table S5.

**Table S6.** The vacancy formation energies and carbon atom migration energies for various nano-carbon materials in the previous reports

| Material | Vacancy formation energy  (Single vacancy) | Vacancy formation energy  (other defect) | Migration energy | reference |
| --- | --- | --- | --- | --- |
| Graphite | 7.4eV |  | 1.7eV (single) | 1 |
| Graphene | 7.5eV | 4eV (double vacancy) | 1.3eV (single) | 2 |
| SWCNT | 4.4~6.8eV |  | 2.2~4.0eV (sinlge) | 3, 4 |
| Fullerene | 3.22~5.58eV | 1.5eV~ 3eV (multi-vacancy) | 2.6~6eV (single)  1.8~2.6eV (double) | 5, 6 |

**Reference**

[1] A. A. El-Barbary, R. H. Telling, C. P. Ewels, M. I. Heggie, P. R. Briddon, Physical Review B, 2003, 68, 144107.

[2] F. Banhart, J. Kotakoski, A. V. Krasheninnikov, ACS nano, 2011, 5, 26-41.

[3] A. V. Krasheninnikov, F. Banhart, J. X. Li, A. S. Foster, R. M. Nieminen, Physical Review B, 2005, 72, 125428.

[4] C.Jin, K. Suenaga, S. Iijima, Nano Letters, 2008, 8, 1127-1130.

[5] D.W. Boukhvalov, M.I. Katsnelson, The European Physical Journal B, 2009, 68, 529-535

[6] H. Hayashi, S. Tamaki, N. Ide, I. Okada, K. Kojima, The Japan Society of Applied Physics, 2002, 41, 6486-6487


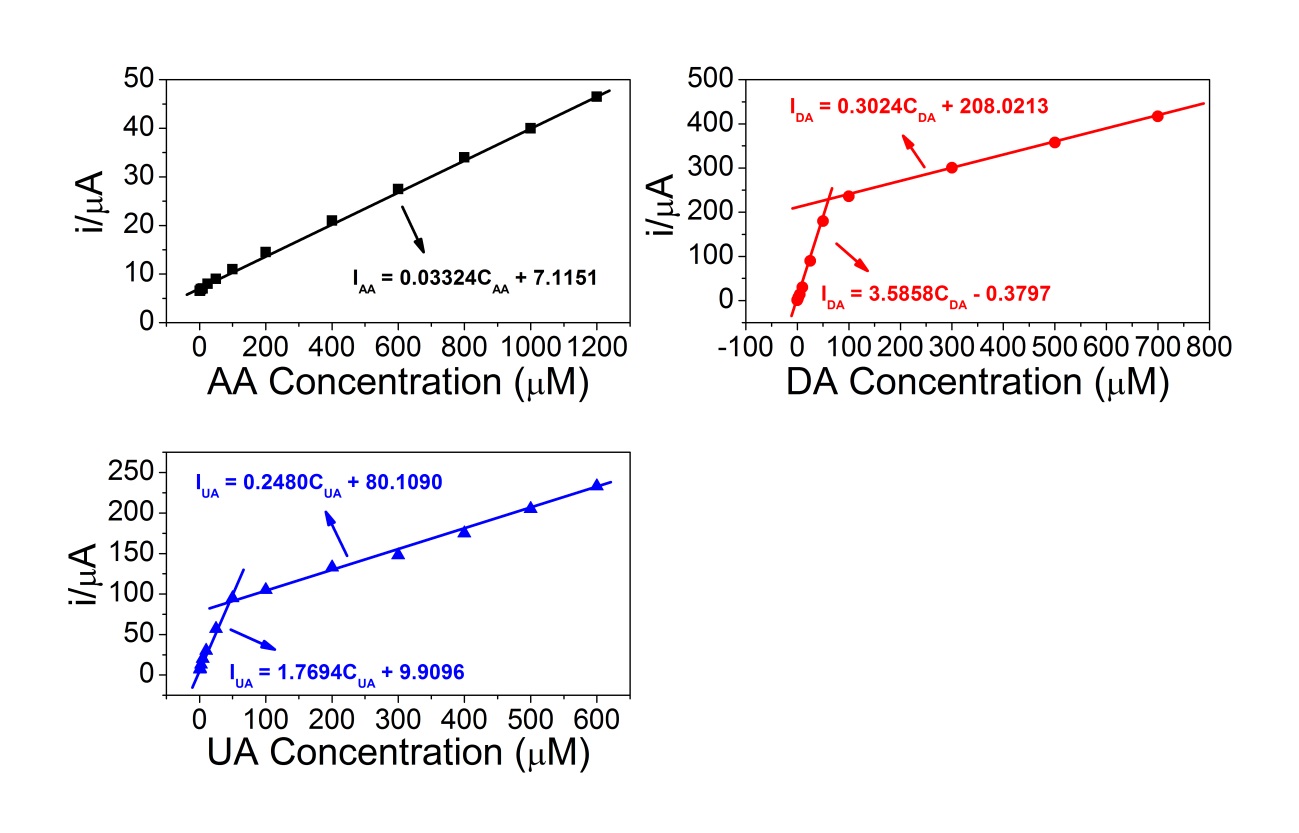


**Figure S19.** The oxidation currents vs concentration profiles obtained from the DPV responses shown in Figure 7

**Table S7**. Analytical parameters for the detection of AA, DA, and UA obtained by DPV using the OLC-1200 electrode

| Analyte | Detection limit  (μM)* | Linear range  (μM) | Linear regression equation  (concentration range) | Correlation factor :r  (concentration range) |
| --- | --- | --- | --- | --- |
| AA | 0.76 | 1-1200 | I_DA_= 0.03324C_AA_+ 7.1151  (1~1200μM) | 0.9986  (1~1200μM) |
| DA | 0.011 | 0.1~700 | I_DA_= 3.5858C_DA_- 0.3797  (0.1~50μM)  I_DA_= 0.3024C_DA_+ 208.0214  (100~700μM) | 0.9981  (0.1~50μM)  0.9988  (100~700μM) |
| UA | 0.036 | 0.5~600 | I_UA_= 1.7694C_UA_+ 9.9096  (0.5~50μM)  I_UA_= 0.2480C_UA_+ 80.1090  (50~600μM) | 0.9901  (0.5~50μM)  0.9936  (50~600μM) |

* The detection limits were calculated by the following equation^1^: Detection limit = 3σ/S (σ: the standard deviation of ten measurements of the blank solution containing interference analytes, S: slope of the linear regression equation for the lower concentration range, Signal to noise ratio = 3)

**Reference**

[1] X.Mao, X.Yang, G.C. Rutledge and T.A.Hatton, ACS Applied Materials & Interfaces, 2014, 6, 3394-3405.

**Statement S5**

As the present oxidation reaction was confirmed to be mixed-controlled, the electron-transfer at the electrode/solution interface must have played an important role, as coupled in series with the analyte diffusion, in determining the oxidation current. On the other hand, the DA and UA molecules are composed of the aromatic rings (benzene ring and purine group), which are strongly attracted to the sp^2^ carbon bonds via the π-π interaction.^1^ Consequently, DA and UA easily adsorb on the OLC surface to form the initial monolayer of adsorption; further adsorption continues but its electron-transfer kinetics keeps being hindered by the presence of the initial adsorption monolayer. By contrast, AA has no aromatic ring so that their adsorption onto the graphene layer is relatively weak. Consequently the AA molecules keep staying at a relatively larger distance from the electrode surface with neither the initial adsorption monolayer formation nor transition to subsequent stage of adsorption; it would accordingly eliminate the gradient transition. It also accordingly suppresses the tunneling probability of the electron from the AA molecule to the electrode surface, relative to DA and UA, resulting in relatively suppressed oxidation current generation.^2^

**Reference**

[1] D. Q. Zhu, J. J. Pignatello, Environ Sci Technol, 2005, 39, 2033-2041

[2] H. Y. Yue, S. Huang, J. Chang, C. Heo, F. Yao, S. Adhikari, F. Gunes, L. C. Liu, T. H. Lee, E. S. Oh, B. Li, J. J. Zhang, T. Q. Huy, N. V. Luan, Y. H. Lee, Acs Nano, 2014, 8, 1639-1646

**Table S8**. Summary of the previous reports concerning the sensitivity of DA detection by DPV, employing various types of nano-carbon electrodes, as compared to that of OLC-1200 electrode in the present study

| Electrode material | Linear regression equation  (concentration range) | Correlation factor (r) | Ref |
| --- | --- | --- | --- |
| Polystyrene-sulfonate -wrapped multiwalled-CNT | I_DA_=0.9576C_DA_+47.9111  (1~150μM) | 0.9947 | 1 |
| N-graphene | I_DA_=0.03195C_DA_-0.0535  (0.5~170μM) | 0.9997 | 2 |
| Pt/MWCNT | I_DA_=3.0768C_DA_+0.8494 | 0.9992 | 3 |
| GONR | I_DA_=3.55C_DA_-94.5 | 0.988 | 4 |
| N-doped porous carbon nanopolyhedra | I_DA_=1.2243C_DA_+4.3641  (0.25~25μM) | 0.9983 | 5 |
| OLC-1200 | I_DA_= 3.5858C_DA_ - 0.3797  (0.1~50μM) | 0.9981  (0.1~50μM) | This work |

**Reference**

[1] R.Manjunathaa, G.S.Suresha, J.S.Melob, S.F.D’Souzab, T.V.Venkateshac, Sensors and Actuators B, 2010, 145, 643-650.

[2] Z.H.Shenga, X.Q.Zhenga, J.Y.Xua, W.J.Baoa, F.B.Wanga, X.H.Xiaa, Biosensors and Bioelectronics, 2012, 34, 125-131.

[3] Z.Dursun, B.Gelmez, Electroanalysis, 2010, 22, 1106-1114.

[4] C.L.Sun, C.T.Chang, H.H.Lee, J.Zhou, J.Wang, T.K.Sham, W.F.Pong, ACS nano, 2011, 5, 7788-7795.

[5] P.Gai, H.Zhang, Y.Zhang, W.Liu, G.Zhu, X.Zhang, J.Chen, Journal of materials chemistry B, 2013, 1, 2742-2749

**Table S9**. Summary of the previous reports concerning the detection limit and linear range of AA, DA, UA without the interference molecules obtained by DPV employing the various types of the nanocarbon electrodes, as compared to those of the OLC-1200 electrode in the present study

| Electrode material | Linear range, μM | | | Measured Detection Limit*  (Calculated Detection Limit), μM | | | Ref |
| --- | --- | --- | --- | --- | --- | --- | --- |
|  | AA | DA | UA | AA | DA | UA |  |
| Pt/MWCNT | 23-880 | 0.043-62 | 0.046-52 | 23  (19.7) | 0.043  (0.0278) | 0.046  (0.032) | 1 |
| 3D N-doped graphene | - | 3-100 | - | - | 3  (0.001) | - | 2 |
| NiO/ZnO | - | 1-100 | 12.9-6290 | - | 1  (0.062) | 12.9  (0.97) | 3 |
| polystyrene/graphene  core-shell microsphere | - | 0.1-20 | - | - | 0.1  (0.02) | - | 4 |
| Gold nanoparticles/Indium Tin oxide-coated glass/reduced graphene oxide | - | 10-1000 | - | - | 10  (0.06) | - | 5 |
| OLC-1200 | 1-1200 | 0.1-700 | 0.5-600 | 1  (0.76) | 0.1  (0.011) | 500  (0.036) | This work |

*We defined the measured detection limit as the lower concentration limit of the linear range of detection.

**Reference**

[1] Z.Dursun, B.Gelmez, Electroanalysis, 2010, 22, 1106-1114.

[2] X.Feng, Y.Zhang, J.Zhou, S.Chen, L.Zhang, Y. Ma, L. Wang, X. Yan, Nanoscale, 2015, 7, 2427-2432

[3] S. Reddy, B. E. K. Swamy, S. Aruna, M. Kumar, R. Shashanka, H. Jayadevappa, Chemical Sensors, 2012, 2, 1-10

[4] S. Li, T. Qian, S. Wu, J. Shen, Chemical Communications, 2012, 48, 7997-7999

[5] J. Yang, J. R. Strickler, S. Gunasekaran, Nanoscale, 2012, 4, 4594-4602

**
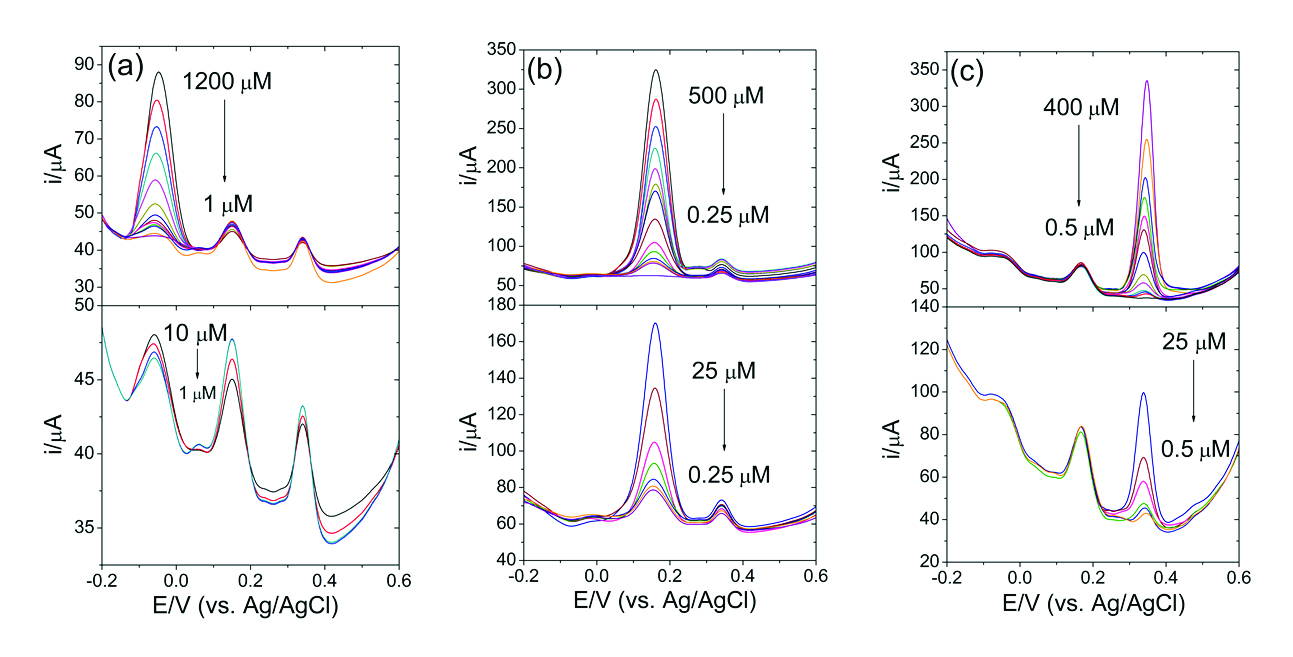
**

**Figure S20.** DPV responses from OLC-1200 electrode for 0.1M PBS containing (a) 1–1200μM AA with 20μM DA and UA, (b) 0.25–500μM DA with 100μM AA and 20μM UA, (c) 0.5–400μM UA with 100μM AA and 20μM DA, respectively.

**Table S10**. Summary of the previous reports concerning the detection limit and linear range of AA, DA, UA with the interference molecules obtained by DPV employing the various types of the nanocarbon electrodes, as compared to those of the OLC-1200 electrode in the present study

| Electrode material | Linear range, μM | | | Measured Detection Limit*  (Calculated Detection Limit), μM | | | Ref |
| --- | --- | --- | --- | --- | --- | --- | --- |
|  | AA | DA | UA | AA | DA | UA |  |
| Polystyrene sulfonate wrapped  multiwall-CNT | 500-2500 | 1-150 | 1-120 | -  (500) | -  (1) | -  (1) | 1 |
| N-graphene | 5-1300 | 0.5-170 | 0.1-20 | 5  (2.2) | 0.5  (0.25) | 0.1  (0.045) | 2 |
| mesoporous carbon nanofiber/pyrolytic graphite | 500-3500 | 4-12 | 2-120 | 500  (50) | 4  (0.02) | 2  (0.2) | 3 |
| GONR | - | 0.5-500 | - | - | 0.5  (0.77) | - | 4 |
| Palladium nanoparticle/graphene/chitosan | 100-4000 | 0.5-200 | 0.5-200 | 100  (20) | 0.5  (0.1) | 0.5  (0.17) | 5 |
| Graphene-SnO2 | - | 0.1-10 | - | - | 0.1  (0.08) | - | 6 |
| N-doped porous carbon  nanopolyhedra | 80-2000 | 0.5-30 | 4-50 | 80  (0.74) | 0.5  (0.011) | 4  (0.021) | 7 |
| OLC-1200 | 1-1200 | 0.25-500 | 0.5-400 | 1  (0.86) | 0.25  (0.030) | 0.5  (0.045) | This work |

*We defined the measured detection limit as the lower concentration limit of the linear range of detection.

**Reference**

[1] R.Manjunathaa, G.S.Suresha, J.S.Melob, S.F.D’Souzab, T.V.Venkateshac, Sensors and Actuators B, 2010, 145, 643-650.

[2] Z.H.Shenga, X.Q.Zhenga, J.Y.Xua, W.J.Baoa, F.B.Wanga, X.H.Xiaa, Biosensors and Bioelectronics, 2012, 34, 125-131.

[3] Y. Yue, G. Hu, M. Zheng, Y. Guo, J. Cao, S. Shao, Carbon, 2012, 50, 107-114

[4] C.L.Sun, C.T.Chang, H.H.Lee, J.Zhou, J.Wang, T.K.Sham, W.F.Pong, ACS nano, 2011, 5, 7788-7795.

[5] X. Wang, M. Wu, W. Tang, Y. Zhu, L. Wang, Q. Wang, P. He, Y. Fang, Journal of Electroanalytical Chemistry, 2013, 695, 10-16

[6] A.Yang, Y.Xue, Y.Zhang, X.Zhang, h.Zhao, X.Li, Y.He, Z.Yuan, Journal of materials chemistry B, 2013,1, 1804-1811

[7] P.Gai, H.Zhang, Y.Zhang, W.Liu, G.Zhu, X.Zhang, J.Chen, Journal of materials chemistry B, 2013, 1, 2742-2749

**
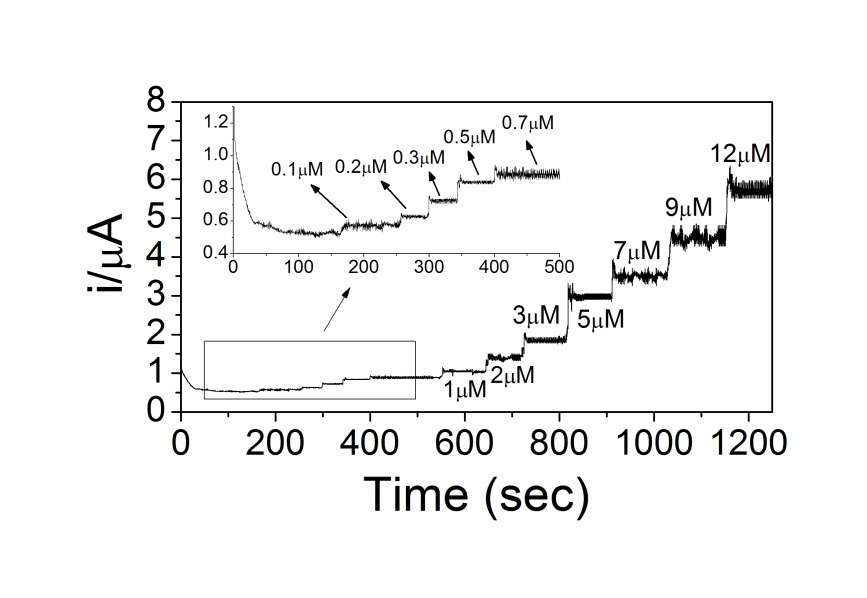
**

**Figure S21.** Chronoamperometric response from the OLC-1200 electrode under a constant potential of 0.2V with the step-wise increase in the DA concentration in the 0.1M PBS solution


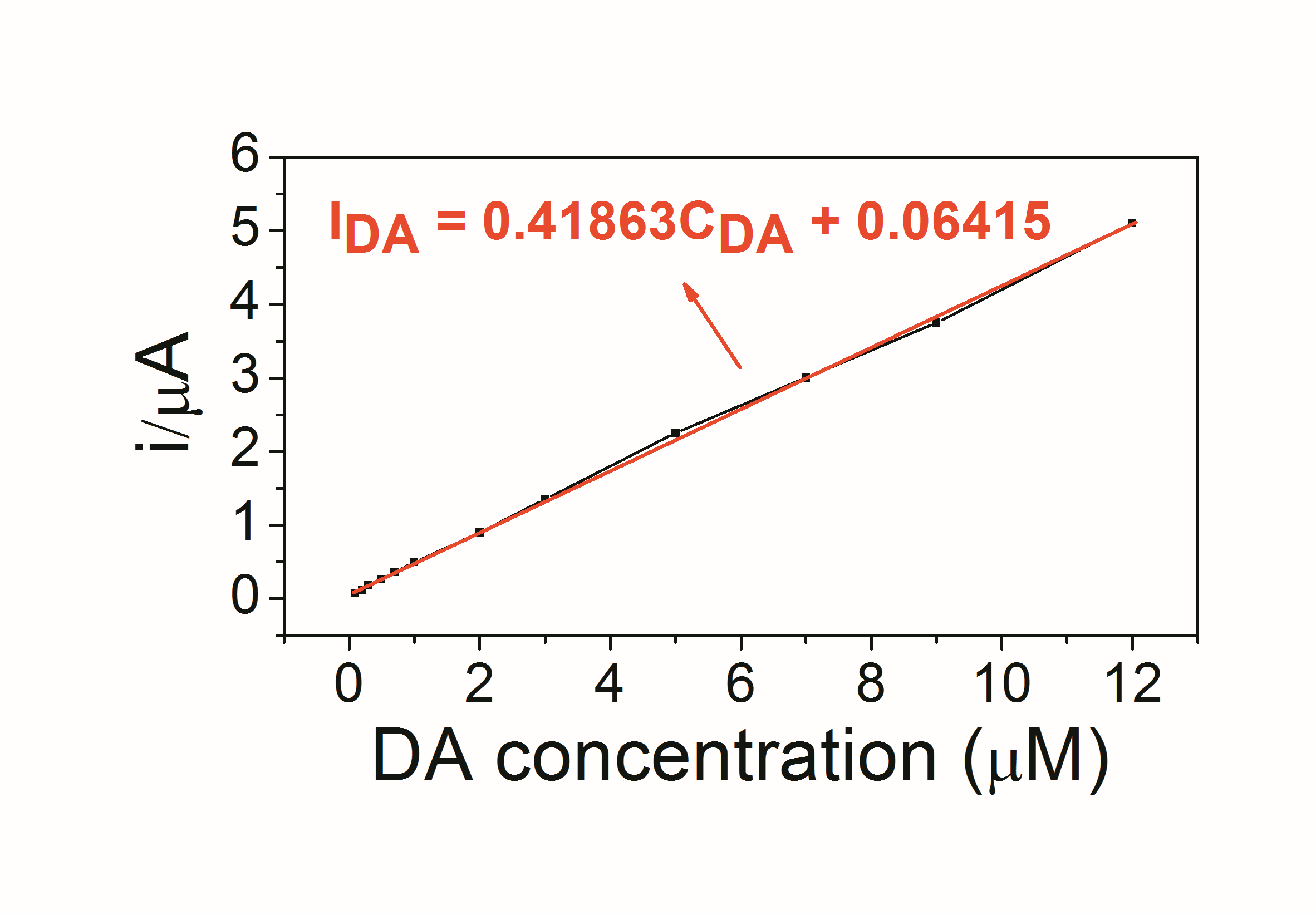


**Figure S22.** The oxidation current (I_DA_) vs DA concentration (C_DA_) profile corresponding to the chrono-amperometric response shown in Figure S21

**Table S11.** Summary of the previous reports concerning the analytical parameters for the DA detection by chrono-amperommetry employing the various types of nano-carbon electrodes, as compared to the OLC-1200 electrode in the present study

| Electrode material | Detection limit (μM) | Linear range  (μM) | Linear regression equation | Response time (sec) | Ref |
| --- | --- | --- | --- | --- | --- |
| Polystyrene sulfonate wrapped multiwalled-CNT | 0.15 | 50~350 | I_DA_ = 12.3702C_DA_ + 97.2152  (r=0.9966) | - | 1 |
| GONR | 0.08 | 0.15~12.2 | I_DA_ = 0.4694C_DA_ + 0.2481  (r=0.9989) | 2.125 | 2 |
| Pt/Graphene | 0.03 | 0.03~8.13 | I_DA_ = 0.9695C_DA_ + 0.0366  (r=0.9976) | 0.4 | 3 |
| OLC-1200 | 0.08* | 0.12~12 | I_DA_=0.41863C_DA_+0.06415  (r=0.9993) | 2.035 | This work |

* The detection limits were calculated by the following equation^4^: Detection limit = 3σ/S (σ: the standard deviation of ten measurements of the blank solution containing interference analytes, S: slope of the linear regression equation for the lower concentration range, Signal to noise ratio = 3)

**Reference**

[1] R.Manjunathaa, G.S.Suresha, J.S.Melob, S.F.D’Souzab, T.V.Venkateshac, Sensors and Actuators B, 2010, 145, 643-650.

[2] C.L.Sun, C.T.Chang, H.H.Lee, J.Zhou, J.Wang, T.K.Sham, W.F.Pong, ACS nano, 2011, 5, 7788-7795.

[3] C.L.Suna, H.H.Leea, J.M.Yanga, C.C.Wub, Biosensors and Bioelectronics, 2011, 26, 3450-3455.

[4] X.Mao, X.Yang, G.C. Rutledge and T.A.Hatton, ACS Applied Materials & Interfaces, 2014, 6, 3394-3405.

**Statement S6**

A similar characterization was carried out on OLC-1200 electrode by chrono-amperometry for the lower concentration range of 0.1-12μM, as shown in Figures S21, S22. The oxidation currents shown in Figure S21 were plotted against DA concentration in Figure S22. At fixed potential of 0.2V, the oxidation current of DA was recorded with the stepwise elevation of the DA. Such analysis enables the real-time monitoring of the analyte concentration, which is impossible in CV or DPV.^1^ In this case, apart from the detection limit, linear range, and sensitivity, an additional important parameter is the response time, all of which were summarized in Table S11. Note that the response time was comparable to that of GONR in the recent report. The limit of detection of OLC-1200 was also comparable to those of other n-C materials shown in the Table S11.


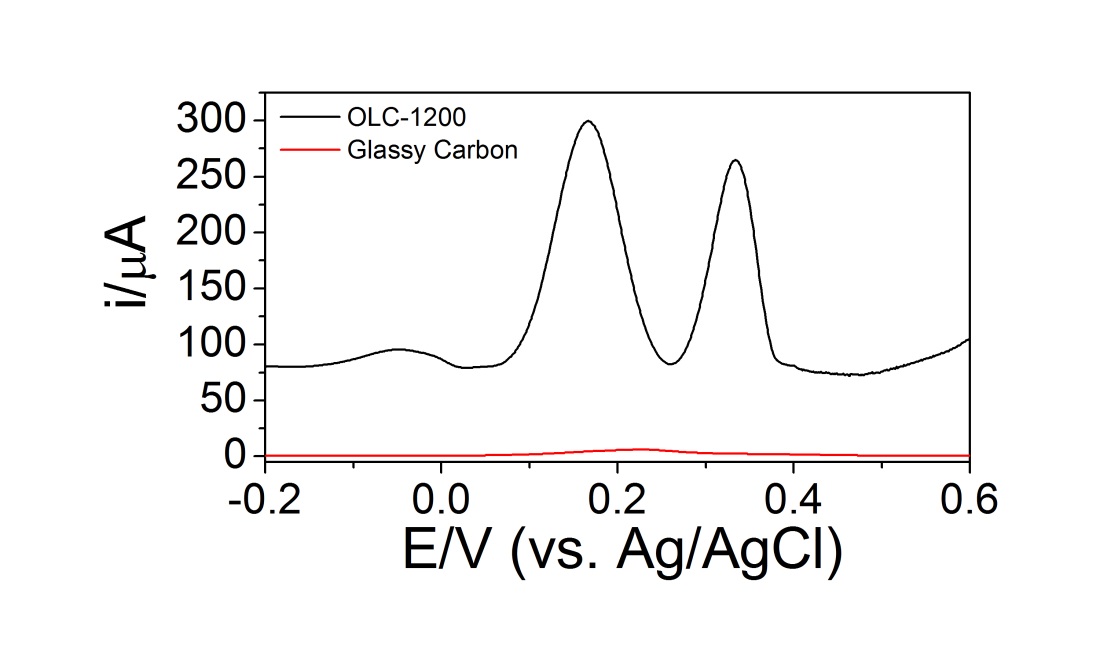


**Figure S23.** DPV responses from the bare glassy carbon electrode, as compared to OLC modified electrode, for the mixture solution containing 0.33mM AA + 0.33mM DA + 0.33mM UA
